# From Polymer to Small Organic Molecules: A Tight Relationship between Radical Chemistry and Solid-Phase Organic Synthesis

**DOI:** 10.3390/molecules16043252

**Published:** 2011-04-18

**Authors:** Danilo Mirizzi, Maurizio Pulici

**Affiliations:** Department of Chemical Core Technologies, Nerviano Medical Sciences S.r.l., V.le Pasteur, 10, 20014, Nerviano (MI), Italy

**Keywords:** radical, solid-phase, SPS, polymer-assisted synthesis, polymer-supported reagents

## Abstract

Since Gomberg’s discovery of radicals as chemical entities, the interest around them has increased through the years. Nowadays, radical chemistry is used in the synthesis of 75% of all polymers, inevitably establishing a close relationship with Solid-Phase Organic Synthesis. More recently, the interest of organic chemists has shifted towards the application of usual “in-solution” radical chemistry to the solid-phase, ranging from the use of supported reagents for radical reactions, to the development of methodologies for the synthesis of small molecules or potential libraries. The aim of this review is to put in perspective radical chemistry, moving it away from its origin as a synthetic means for solid supports, to becoming a useful tool for the synthesis of small molecules.

## Abbreviations

AIBN: Azo-bis-isobutyronitrile
AMBN: azobismethylisobutyronitrile
ATRC: Transition-metal-catalyzed atom transfer radical cyclization
9-BBN: 9-Borabicyclo[3.3.1]nonane
CAN: Ceric ammonium nitrate
DAST: Diethylaminosulfur trifluoride
DCE: 1,2-Dichloroethane
DCM: Dichloromethane
DIC: Diisopropyl carbodiimide
DIPAD: Diisopropyl azadicarboxylate
DIPEA: diisopropylethylamine
DLP: lauroylperoxide
DMA: *N,N*-Dimethylacetamide
DMAP: 4-dimethylaminopyridine
DMF: N,N-Dimethylaminoformamide
EPHP: N-ethylpiperidine hypophosphite
FRET: Fluorescence Resonance Energy Transfer
HASC: α-Hetero-Atom Substituted Carbonyl
HBTU: 2-(1H-Benzotriazole-1-yl)-1,1,3,3-tetramethyluronium hexafluorophosphate
HMPA: Hexamethylphosphoramide
IBX: 2-Iodoxybenzoic acid
LA: Lewis acid
NMM: N-methyl morpholine
NMP: N-methyl pyrrolidine
NMQ: N-methylquinolinium
PMHS: Polymethylhydrosiloxane
RCM: Ring-closing metathesis
SET: single electron transfer
SP: Solid-phase
SPS: Solid-phase synthesis
TBGH: Tributylgermanium hydride
TBTH: Tributyltin hydride
TEMPO: Tetramethyl pentahydropyridine oxide
TFA: Trifluoroacetic acid
THF: Tetrahydrofuran
TON: Turnover number
TTF: Tetrathiofulvalene
TTMSS: Tris(trimethylsilyl)silane
Troc: 2,2,2-Trichloroethoxycarbonyl

## 1. Introduction

Since Gomberg’s discovery of free radicals as chemical entities [1], the interest around this electron-deficient species has increased throughout the years. Their success arrived during WWII, when radical chemistry took a primary role in the manufacturing of synthetic substitutes of natural rubber. Nowadays, radical processes are used to prepare 75% of all polymers, including the solid supports employed in organic synthesis.

An additional and interesting application of radical chemistry involves post polymerization modifications, which transforms the surface of the polymer for several purposes [2,3,4]. The change of polymers’ surface characteristics allows the acquisition of new properties of the solid phase (SP) and, although this represents an interesting scientific aspect of the subject, touching the field of organic synthesis as well, it is also somewhat out of the purpose of this review and, for those interested, it would be more appropriate to refer to more specific literature.

Inevitably, all of these roles of radical chemistry have allowed creating a thread with SP synthesis. SP synthesis is an important feature in modern organic chemistry, however, the role of free radicals includes more than the methodologies just used for polymerization. The real boost came after physical organic chemists begun to determine absolute rates of radical reactions [5,6], together with the advent of new spectroscopic techniques (ESR) to detect them. At that point, radical chemistry became a more and more common methodology for day-to-day organic synthesis. With a wider range of available radical reactions, several applications in the synthesis of natural compounds, drug-like molecules and functional groups interconversion strategies were identified. It would have been just a matter of time to find extensions of those methodologies to SP approaches.

This review focuses on the applications of radical chemistry in solid-phase synthesis of single or classes of compounds, which includes also resin loading and cleavage and the use of solid-supported reagents. This subject has been last reviewed a few years ago [7]. The aim of this work is not to offer a comprehensive overview of the applications of radical chemistry on SP, but rather to emphasize the methodologies and developments in a field that might be useful in daily organic chemistry.

However, since classical radical chemistry is governed by kinetic considerations, before going through the examples that the literature offers on the subject it is fundamental to understand how radical reactions behave when they take place on the surface of a solid bead. In this respect it is important to mention Curran’s work [8], dealing with the measurement of the kinetics of radical reactions on SPs. Curran calculated the rates of common reactions involving radical precursors anchored to an Ellman THP resin by the ratio of the possible products, in relation to the same reaction run in solution.

He found that the rate constant of *H*-abstraction from Bu_3_SnH by an alkyl radical linked to SP is probably not so different from the solution-phase one. This means that reactions of polymer-bound radicals, at least those mediated by tin hydride, can be planned as if they were performed in solution.

On the other hand, experiments performed with the generation of aryl radicals demonstrated that Ellman’s resin behaves like a solvent with relatively reactive C-H bonds. This means that *H*-abstraction from the polymer backbone is a feasible process, implicating that, at least in the case of the studied aryl radical, it is difficult to set up reactions whose kinetics are slow. Accordingly, for resin-bound aryl radicals there is a limitation to the fastest intramolecular classes, while slower intra- and bimolecular reactions will suffer from competing hydrogen transfer from the backbone and linker C-H bonds.

## 2. Solid-Supported Reagents, Resins and Linkers

Among the various relationships between radical chemistry and SP, two interesting aspects are the use of special resins or linkers cleavable under radical conditions and solid-supported reagents designed to assist radical reactions in solution. In this regard, this section will provide a review of the literature.

### 2.1. Linkers and Resins

In the quest for reliable, clean and flexible linkers for SP synthesis, an important role is assigned to those cleavable *via * radical mechanisms. In particular, photolabile moieties represent the great majority of the type and several resins with appropriately designed linkers are commercially available. Since this specific subject covers more than 20 years of research and has been accurately review in the past, we refer readers to more appropriate publications [9,10].

Beside light-sensitive linkers, a number of studies have treated innovative linkages based on cleavage under radical conditions. Radical cleavage of linker units usually enables the introduction of either a hydrogen atom at the cleavage site (traceless linker [11]), or, which is of even more relevance in the context of diversity oriented synthesis [12], of an additional diversity element (diversity linker unit [13]). In this section of the review, the attention will be shifted among the “key” atom in the linker.

#### 2.1.1. Sulfur-Based Linkers

**Scheme 1:**
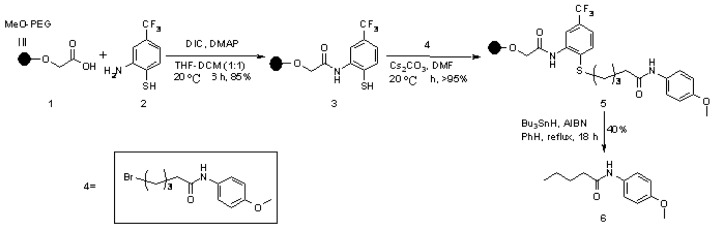
Janda’s sulfur-based linker.

In the need of new methods for SP synthesis with *C-H* bond formation after cleavage (traceless linker strategy), a linking strategy consisting in the presence of a sulfur atom, in different oxidation states susceptible of radical cleavage, was developed. The work performed by Janda [14] falls under this category (Scheme 1).

Loading of test compound **4** was performed on a soluble PEG-polymer using the pre-loaded linker **3**. Reductive cleavage with C-H bond formation was tried under classical radical conditions; however, hydrogenolysis (Ni-Raney) gave better yield (94%) after shorter reaction time (3 h).

**Scheme 2:**
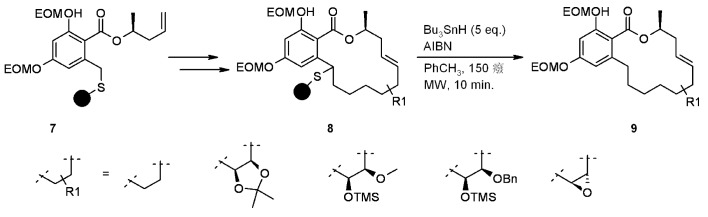
Winssinger’s sulfur-based linker.

In the following example, Winssinger and coworkers [15] completed the synthesis of a series of derivatives of the natural product aigialomycin D, in order to explore the potential activities of this class of compounds (Scheme 2). Polymer-supported **8** was generated from **7** through functionalization of the benzyl carbon and subsequent RCM. Cleavage from the resin under radical conditions gave reduced products **9**.

**Scheme 3:**
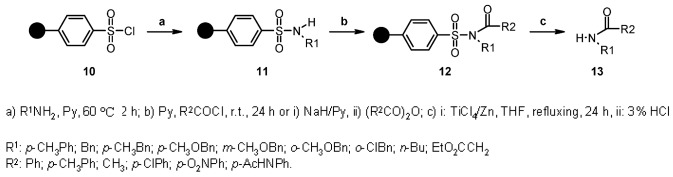
Huang’s sulfur-based linker.

Radical desulfonation is a known reaction in solution-phase synthesis [16]. Based on this reaction, Luo and Huang [17] studied the potentiality of sulfonamide moieties as traceless linkers cleaved by radical mechanism in order to generate a library of secondary amides (Scheme 3).

**Scheme 4:**
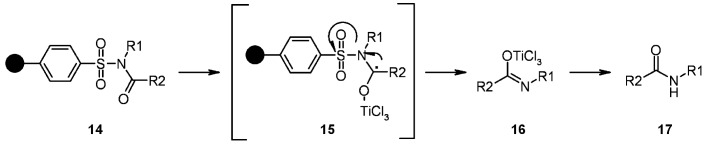
Huang’s proposed mechanism.

The results of the final cleavage were deeply dependent on the nature of the R^1^ group. Generally speaking, substituents containing electron-donating groups (such as a *m*-methoxyphenyl group) were cleaved successfully, while those containing electron-withdrawing moieties, such as a nitrophenyl group, gave no cleavage at all. In the former case, the presence of *o,p*-methoxyphenyl groups gave by-products from a competitive radical cascade, while, in the latter, reduction and acetylation of the resulting amino group were necessary to perform radical cleavage. The proposed mechanism (Scheme 4) gives an idea about how the nature of R^1^ can influence the radical fragmentation. In fact, if R^1^ contains a carbon atom capable to form a stable radical, the direction of the fragmentation can vary from the one illustrated, yielding unwanted side-products.

**Scheme 5:**
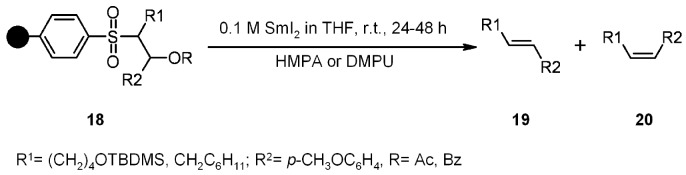
De Clercq’s proposed mechanism.

D’Herde and De Clercq [18] used the single-electron reductive property of samarium(II) for the cleavage of PS-β-benzolyloxysulfones in a Julia-Lythgoe olefination, where the SP-sulfone partly works as a traceless linker (Scheme 5). The best E:Z ratio (**19**:**20** = 94:6) was obtained when R = Bz and 1,3-dimethyl-3,4,5,6-tetrahydro-2(1H)-pyrimidinone (DMPU) was used as an additive, although isolated yields were rather low (around 25%).

**Scheme 6:**
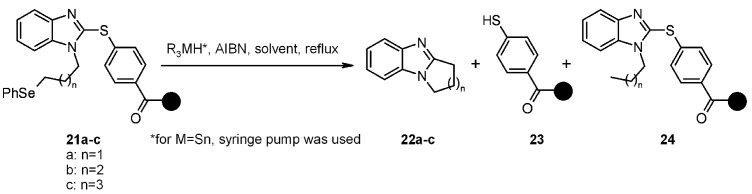
Bowman’s sulfur-based linker.

Radical cyclization to prepare azoles has been explored in solid-phase synthesis using a thiophenyl linker [19], using benzimidazole as a probe heterocycle. To prove the validity of the methodology, the same reactions were also carried out in solution (Scheme 6). Radical precursors **21a-c** were loaded on the resins through the acid moiety on the arylthiol.

**Scheme 7:**
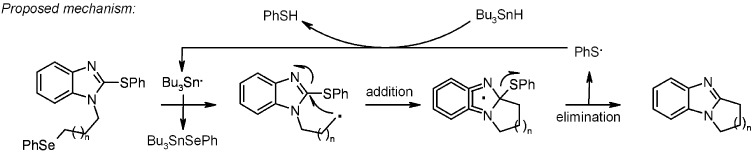
Bowman’s proposed mechanism.

For n = 1, under the best conditions, only a 11% of desired product **22a** was observed, in line with the results obtained in solution, indicating that this 5-*exo*-trig cyclization is not strongly favored. For n = 2 the reaction was smoother and use of TBTH gave a 60% of isolated **22b**. When TBGH was used, a lower yield of **22b** was isolated (22%), while TTMSS gave similar results (20%). For n = 3, again poor yields were obtained (4%), just as the reaction run in solution with TBTH. Three resins were used for the scope, however, if Wang and Rink resins gave similar results, amino-Merrifield proved to be unsuitable, as only traces of desired products were found (for n = 1). The rationale behind the use of 2-thioaryl derivative is summarized in the proposed mechanism (Scheme 7).

The 2-thiophenyl group behaves as a radical leaving group to achieve rearomatization and, subsequently, as radical carrier to abstract a hydrogen atom from the metal hydride, propagating the chain [20]. Following the mechanism, it is possible to note that the desired cyclized product **22** is released after formation, while side-products, such as non-cyclized, reduced **24** and unreacted starting material are left on the resin.

In order to shorten the reaction times, focused microwave irradiation was also used. As a test case radical precursor **21b** (n = 2) on Wang resin was taken. Under optimized conditions (using AMBN) a 52% yield was obtained, very close to the 60% obtained under conventional heating, but with dramatically shortened reaction times (20 min *vs.* 7 h). The reaction was also run on **21a** (n = 1) on amino-Merrifield resin, giving poor yields (3% of **22**).

**Scheme 8:**
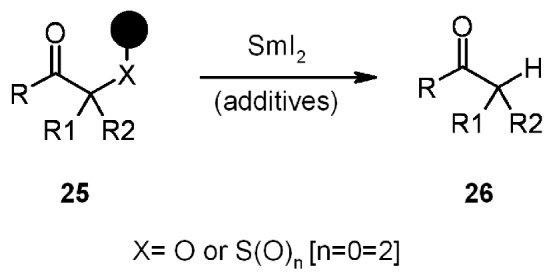
Procter’s HASC strategy.

Among the new SP techniques, the α-Hetero-Atom Substituted Carbonyl (HASC) traceless linker studied by Procter, occupies an important role. It exploits the SET characteristics of Sm(II) for reductive cleavage of an X-C bond. The general idea of the methodology starts with the attachment of α-halo-carbonyl compounds to an appropriate resin, followed by further transformation of the resin-linked compounds, ending in the reductive cleavage from the resin (Scheme 8).

**Scheme 9:**
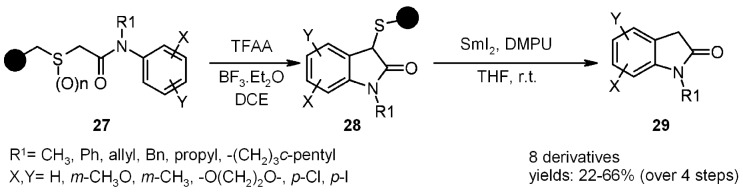
Procter’s use of a sulfur linker for the synthesis of oxindoles.

**Scheme 10:**
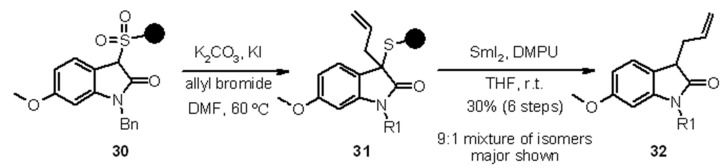
Procter’s synthesis of 3-allyl oxindoles.

Heterocycle synthesis was performed following this strategy using a sulfur-linkage [21,22,23]. Benzylthiol resin **27** was prepared from Merrifield resin, treated with the appropriate α-bromoacetamide and oxidized (Scheme 9). This activated substrate was then submitted to the Pummerer cyclization to yield oxindole **28**, which could be cleaved using SmI_2_, giving the final products **29** in good yield, or alternatively, it could be further functionalized with allyl bromide prior to detachment (Scheme 10).

**Scheme 11:**
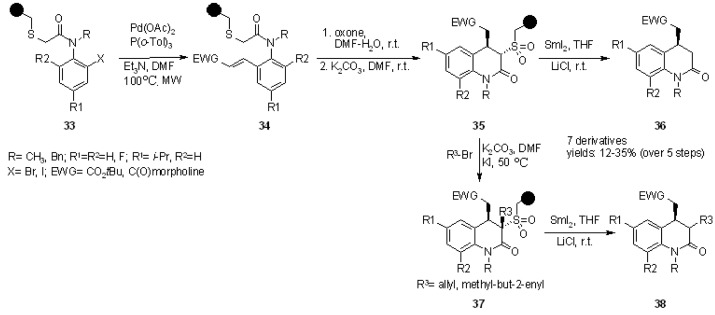
Procter’s use of a sulfur linker for the synthesis of tetrahydroquinolones.

Tetrahydroquinolones were prepared in a similar fashion, using a Heck reaction followed by sulfur oxidation and Michael addition to perform cyclization. A couple of examples are shown in Scheme 11.

Reductive cleavage from the resin was performed before further functionalization to give **36** or the sulfur-bearing carbon atom was derivatized and only subsequently cleaved off the resin to give **38**.

**Scheme 12:**
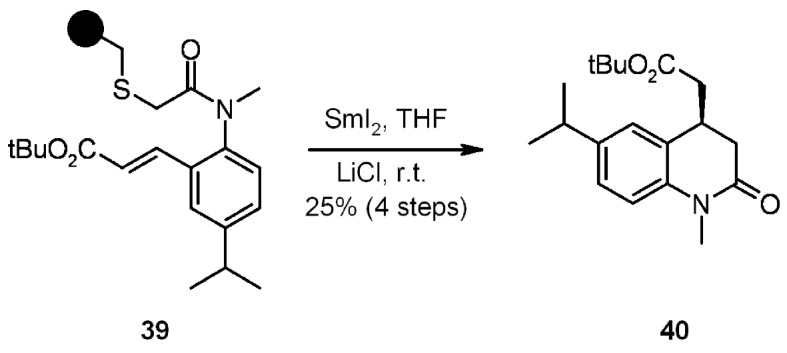
Example of SmI_2_-mediated cyclative cleavage.

In Scheme 12 an example of samarium iodide-mediated cyclative cleavage to give **40** through a radical or ionic process is shown.

**Scheme 13:**
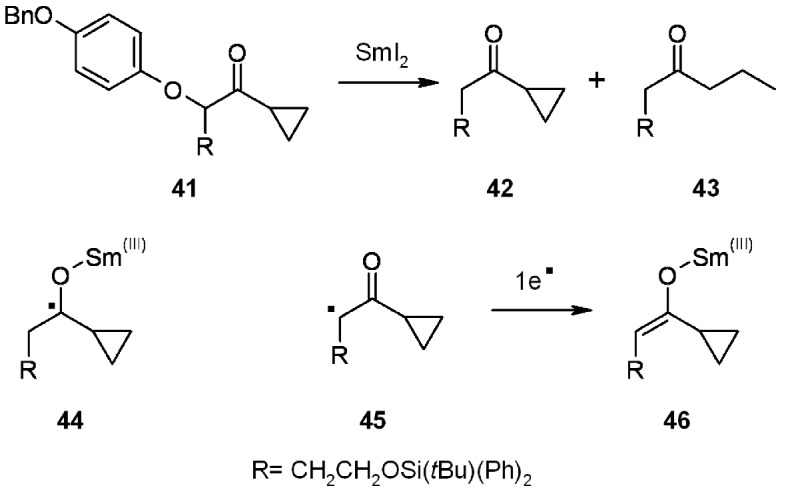
Procter’s proposed mechanism.

The general mechanism for samarium-induced reductive cleavage of α-heteroatom carbonyl compounds is displayed in the scheme below (Scheme 13).

The cleavage mechanism was studied in solution for cyclopropyl derivative **41**. The samarium SET reductive cleavage would account for a radical type **44**, leading to cyclopropyl ring opening (compound **43**) *via* a cyclopropylmethyl radical. However, since there was no trace of ring-opened derivative **43**, the alternative and more probable explanation accounts for different intermediates such as **45**-**46**. The presence of a samarium enolate of the kind of **46** was demonstrated by the capturing of electrophiles during cleavage.

#### 2.1.2. Selenium (and Tellurium)-based Linkers

A number of reports deal with the use of selenium linkers, which are known to be amenable to several cleavage strategies, and the topic has been reviewed recently [24]. The following section is simply meant to highlight the value of such types of linkers in contemporary SPS.

Ruhland and coworkers produced interesting works replacing sulfur with selenium, which proved to be a versatile traceless linker in both aliphatic C-H bond-forming reactions, taking advantage of easier homolytic cleavage, and alkene formation by selenium oxidation followed by β-elimination. In a first paper [25] bromopolystyrene **47** was obtained through thallium acetate-catalyzed bromination of commercially available polystyrene.

**Scheme 14:**
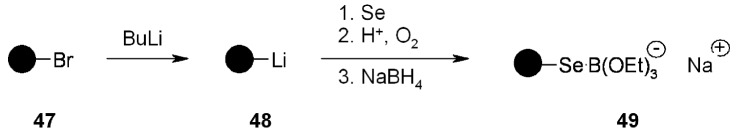
Ruhland’s synthesis of selenium-based resin.

**Scheme 15:**
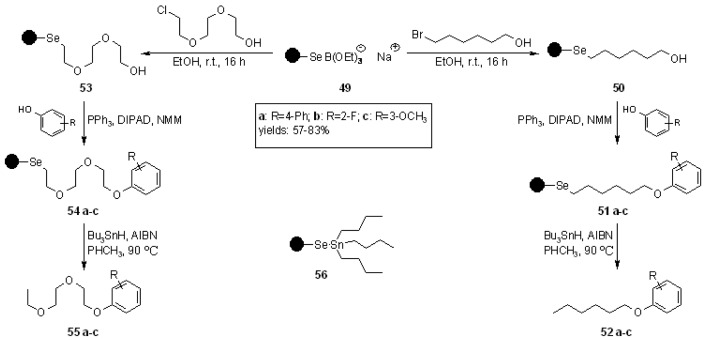
Ruhland’s synthesis of ethers library.

After lithiation with BuLi, the resin was treated with selenium, in order to replace the metal anion. Reduction with NaBH_4_ was necessary because of the occurrence, during work-up in the presence of air (step 2) of Se-Se bridges. Reduction gave back the swelling properties to the resin, lost because of the high degree of cross-linking encountered in step 2. Loading of resin **49** resulted lower compared to the bromopolystyrene **47**, probably due to incomplete reaction with selenium or partial availability of the metal in the resin itself. To prove the utility of resin **49**, it was carried out the synthesis of an alkyl-aryl ether small library using the Mitsunobu reaction (Scheme 15).

After application of the Mitsunobu protocol, cleavage was performed under radical conditions. Contrary to the reaction carried out in solution, the tributylstannyl phenyl selenide **56**, often a problem for efficient purification, remained anchored on the SP and the only by-product of the cleavage was the excess Bu_3_SnH, which was efficiently separated by SP extraction. 

Polymer-bound tellurium was next introduced by the same group [26], which was prepared exploiting a strategy similar to the above. Also in this case a small library based on the Mitsunobu reaction was produced, following tributyltin-mediated product detachment. Interestingly and contrary to expectations, it was found that the reactivity of the polystyrene-bound tellurium towards homolysis is lower in comparison to the polystyrene-bound selenium. This is possibly due to residual elemental tellurium (remaining within the resin) interfering with the radical chain process in the cleavage step.

**Scheme 16:**
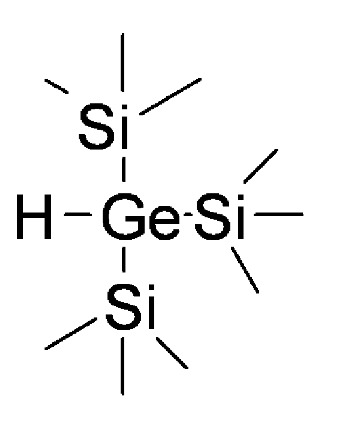
Tris(trimethylsilyl)germane.

The selectivity of bond cleavage offers an important general advantage when utilizing this SP methodology. However, contamination of the cleaved products by tin derivatives can hold back its use, especially for biologically interesting libraries. To address this issue the same group showed later that it is possible to use greener traceless cleavage, exploiting silanes or germanes as valid alternatives to tin-containing reagents. In fact, it was demonstrated [27] that among others especially tris(trimethylsilyl)silane, performs similarly to tributyltin hydride in homolytically cleaving alkyl-selenium derivatives. On the other hand, resin-bound tellurium appears to have a preference for tris(trimethylsilyl)germane (see Scheme 16). In general, however, both yield and purity are superior for the polystyrene-bound selenium, though tellurium is somewhat less sensitive to the number of cleavage reagent equivalents.

**Scheme 17:**
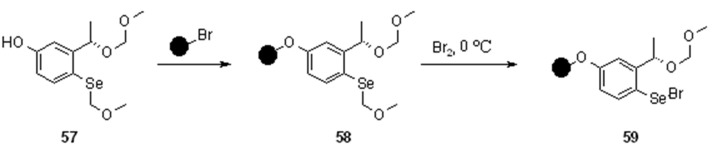
Synthesis of asymmetric selenyl linker.

**Scheme 18:**
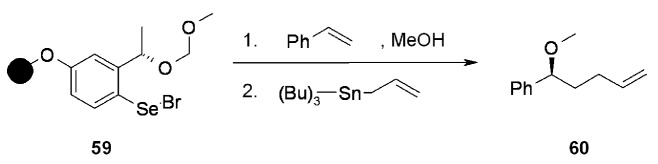
Application of asymmetric selenyl linker.

A chiral selenium electrophile has been also developed and applied to the stereoselective selenenylation of alkenes. The linker has been generated on solid-phase by loading a methoxymethyl-protected selenophenol to a suitable polymeric support, and subsequently generating the electrophile by reaction with bromine at low temperature (Scheme 17). Indeed, mesoporous silica proved superior to other supports, although its loading is usually low. Polystyrene works better than TentaGel and it was predominantly used for the stereoselective additions. Selenenylation of alkenes in the presence of a suitable nucleophile, usually an alcohol, yielded up to 80% ee, making the polymer-supported reagent almost equally attractive as its soluble counterpart (Scheme 18).

Different cleavage protocols were shown in the paper, including the classical oxidation followed by β-elimination, tributyltin hydride-mediated homolytical carbon-selenium bond cleavage and also the use of allyltributyltin, which led to the introduction of an allyl moiety in the product (Scheme 18).

**Scheme 19:**
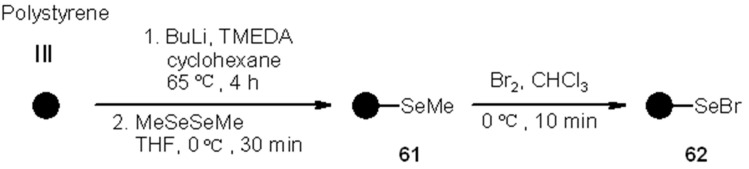
Nicolaou’s preparation of PS-selenyl bromide.

Supported selenyl bromide was prepared also by Nicolaou and coworkers [28] and showed its utility, beside that of a series of other selenium-containing resins, for the use in combinatorial chemistry as linkers (Scheme 19).

**Scheme 20:**
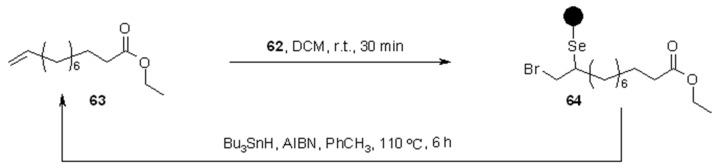
Nicolaou’s SP alkene selenylation.

Compound**62** was exploited to load alkene **63**, and subsequently cleaved under radical conditions (Scheme 20).

**Scheme 21:**
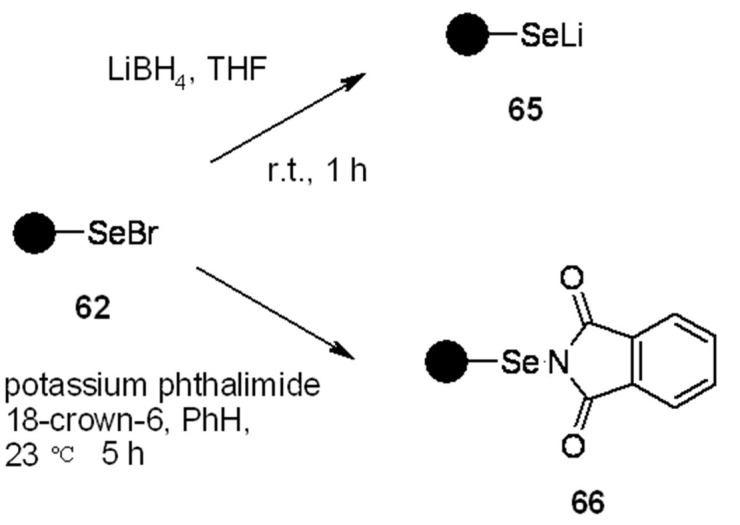
Nicolaou’s modification of PS-selenyl bromide.

Compound **62** can also be reduced to resin **65** (Scheme 21), which can be reacted with halides to load the corresponding alkane. Standard cleavage under radical conditions gave reduction product **69** (Scheme 21).

**Scheme 22:**
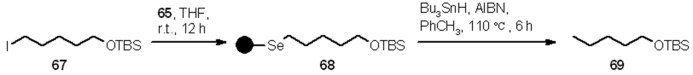
Nicolaou’s use of resin **65**.

**Scheme 23:**
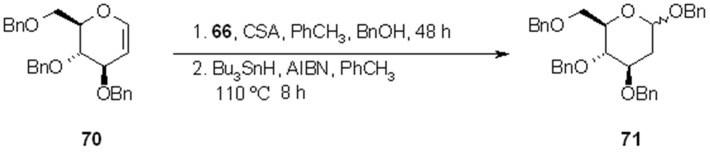
Nicolaou’s use of resin **66**.

In addition, the same resin **62** can give the phtalimido-derivatives **66**, used for insertion of benzyl alcohol (product **71**) onto the alkene moiety (**70**), after radical cleavage (Scheme 23). 

**Scheme 24:**
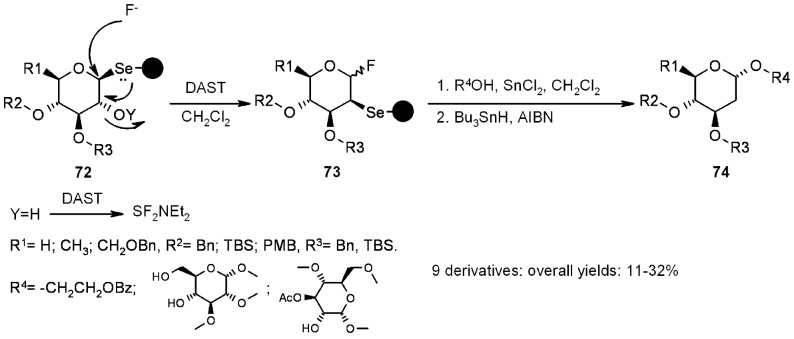
Nicolaou’s synthesis of 2-deoxy glycoside library.

In another interesting work, the same group [29,30] used selenium-based linkers, followed by reductive radical cleavage from the resin, for the synthesis of a small library of 2-deoxy glycosides (Scheme 24).

**Scheme 25:**
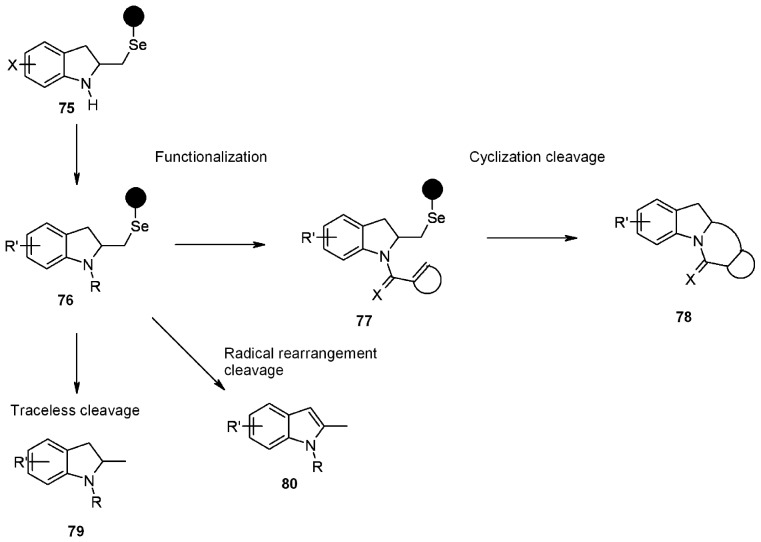
Nicolaou’s SP selenium-based synthesis of heterocycles.

A small library of 3 × 3 was generated (3 “donors”-the sugars described by R^1^, R^2^ and R^3^- and 3 “acceptors”, described by R^4^), with overall yields between 11-32%. The key reaction was the 1,2-seleno migration promoted by diethylaminosulfur trifluoride (DAST), while radical cleavage was typical of selenium-based linkers. Selenium-functionalized resins were exploited by Nicolaou’s group [31,32] also in the studies towards the synthesis of heterocycles, namely indolines, indoline-based policyclic systems and indoles, which were obtained *via* different mechanisms of radical cleavage (Scheme 25).

**Scheme 26:**
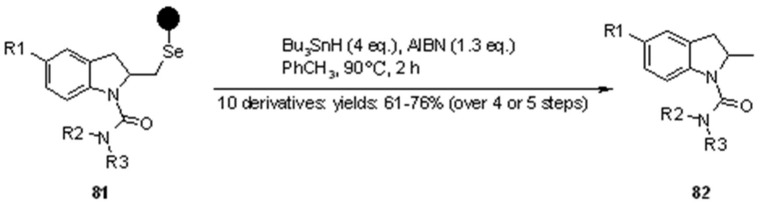
Nicolaou’s selenium traceless linker strategy.

Starting from solid-supported selenyl bromide, indolines **75** were prepared by Lewis-acid mediated cycloloaddition using the appropriate *o*-allyl anilines. Subsequent functionalization at the nitrogen atom and/or at the substituent on the benzene ring allowed the introduction of additional diversity elements. Use of different cleavage strategies enabled then the preparation of small libraries of drug-like molecules. This represents an interesting extension of this methodology to combinatorial chemistry for the synthesis of bioactive compounds. In Scheme 26 a classical traceless cleavage is shown. The mechanism entails the homolytic cleavage of the Se-C bond, therefore generating a carbon-centred radical, quenched by the hydrogen-donor Bu_3_SnH. 

**Scheme 27:**
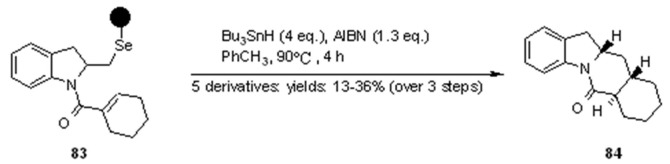
Nicolaou’s synthesis of tetracycle **84**.

In the cyclization cleavage shown in Scheme 27, formation of a carbon-centred radical was exploited by using an appropriate alkene moiety on the nitrogen side-chain. Radical addition/cyclization was then followed by quenching of the resulting radical by tin hydride. The products of the reactions were polycyclic indoline derivatives, with only one diasteroisomer isolated.

**Scheme 28:**
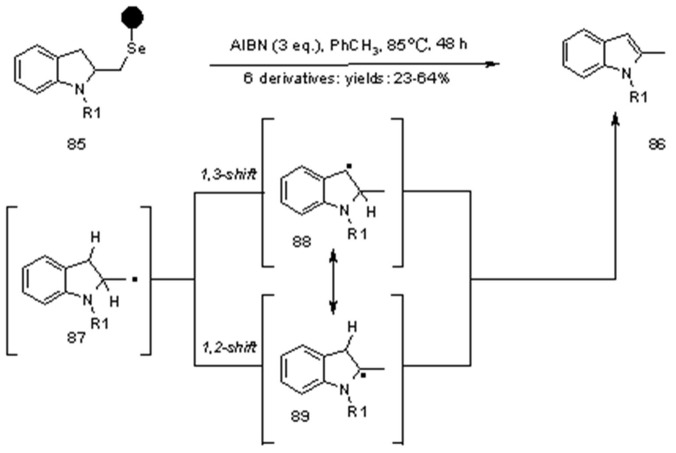
Nicolaou’s *C*-centred radical shift.

An example of cleavage by radical rearrangement is shown in Scheme 28. In this case, the lack of *H*-donor makes the *C*-centred radical enough long-living to perform a shift. Although the nature of the shift is unknown, any of the supposed intermediates radical **88** and **89** can undergo oxidation to the indole ring by loss of a hydrogen atom.

**Scheme 29:**
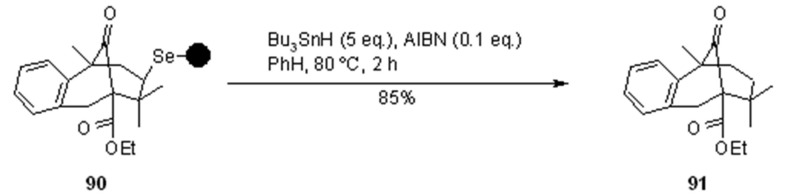
Nicolaou’s synthesis of bicycle **91**.

**Scheme 30:**
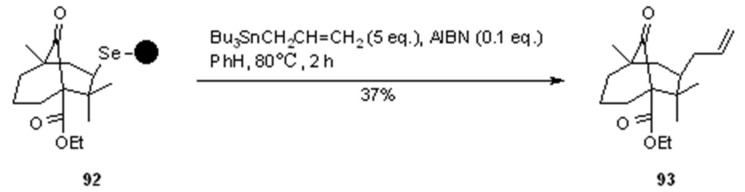
Nicolaou’s synthesis of bicycle **93**.

Very interesting are also other examples from the same group [33], which used the same cycloloadition/radical cleavage methodology to construct [3.3.1]bicycles from properly designed substrates (Scheme 29 and Scheme 30).

As before, bicycles **90** (Scheme 29) and **92** (Scheme 30) were prepared by electrophilic cyclization with a solid-supported-SeBr resin, while cleavage was performed in a traceless manner (Scheme 29) or by trapping the resulting *C*-centred radical with allystannane (Scheme 30).

Although the methodologies shown above proved efficient for the synthesis of small compound libraries, it is important to highlight a major drawback that is implicit in the cleavage mechanism. In fact it was necessary to separate the desired products from the residues of the radical reaction, an option often tedious and difficult that biases the use of toxic tin derivatives for in-solution radical chemistry.

**Scheme 31:**
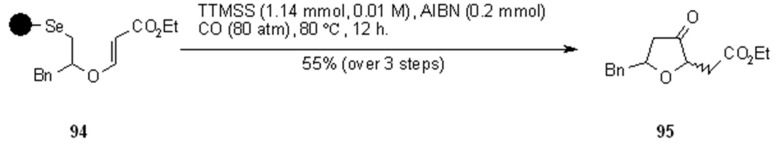
Engman’s use of a selenium linker.

Another interesting cleavage methodology was reported by Engman and coworkers [34], who studied the radical carbonylation/cyclization to make tetrahydrofuran-3-ones. The method was first developed in solution, exploiting alkyl phenyl selenides and then applied to a selenium-based resin **94**, prepared in a few steps from cross-linked (1%) polystyrene (Scheme 31).

TTMSS was chosen instead of the more common TBTH, which gave a large amount of reduced (non-cyclized) product, whereas, increasing the concentration of the H-donor resulted in poorer yields of the desired *vs.* reduction product. TBGH) gave worse results compared to TTMSS. Lower carbon monoxide pressure gave also more product of reduction. The final product **95** was isolated as a 9:1 mixture of cis/trans isomers (55% yield calculated over 3 steps, 2 of them for the synthesis of **94**).

**Scheme 32:**
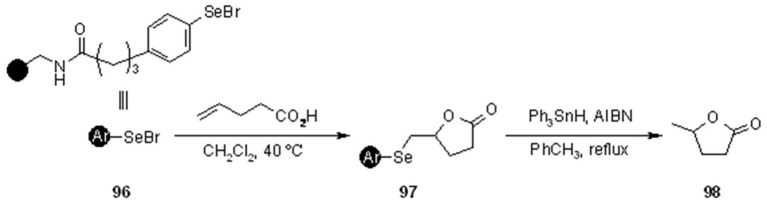
Fujita’s SP selenolactonization of 3-butenoic acids.

Fujita *et al.* [35] used PS-arylselenides for the selenolactonization on 3-butenoic acids with successive cleavage via radical reduction (Scheme 32). The yield of the process to give **98** was rather low (20%), a result explained by Ph_3_SnH-promoted side-reactions with the amide linker.

**Scheme 33:**
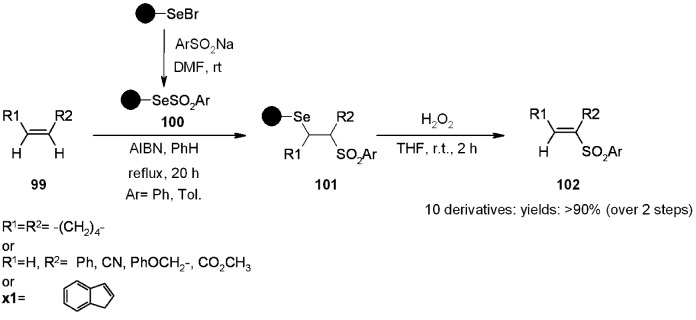
Huang’s synthesis of selenosulfonates.

Polystyrene-supported selenosulfonates can be conveniently prepared from the easily accessible selenobromide by reaction with arylsulfinates. Qian and Huang [36] studied the radical addition of such PS-selenosulfonates to alkenes, with subsequent oxidation-elimination of selenium to obtain vinyl sulfones (Scheme 33).

**Scheme 34:**
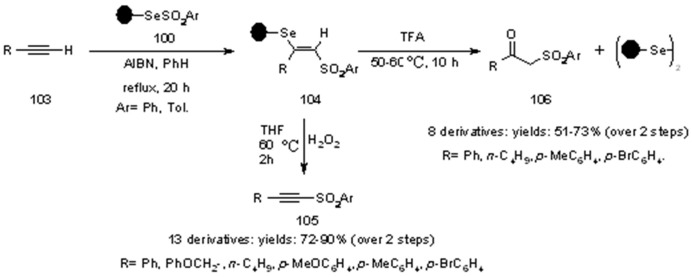
Huang’s synthesis of acetylenic sulfones and β-keto sulfones.

Radical addition was performed under classical radical conditions and the overall yields after cleavage were rather high. The same group also performed the radical addition of PS-arylselenosulfonates to alkynes, followed by oxidation to selenoxide and *syn*-elimination to give acetylenic sulfones **105** [37] or by acidic hydrolytic cleavage from the SP, for the preparation of β-keto sulfones **106** [38] (Scheme 34).

Cleavage under oxidation-elimination conditions (to give **105**) gave higher yields compared to the acidic cleavage (to give **106**), as shown. In any case, there was practically no need for further purification after cleavage from the resin and the PS-diselenide obtained after acidic cleavage could be regenerated.

**Scheme 35:**
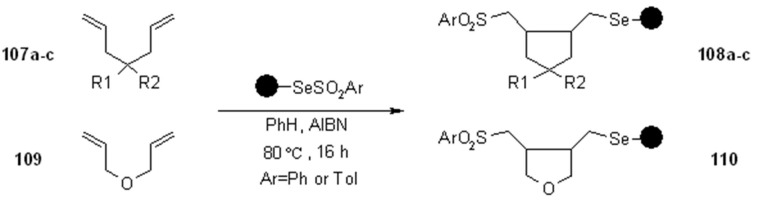
Huang’s SP-atom transfer cyclization.

In another interesting work, a solid-supported selenosulfone as radical transfer agent [39]. In this case however, the reaction led to a cyclization product that, therefore, remained anchored to the SP. Only successively this was cleaved form the resin by the classical oxidation-elimination protocol (Scheme 35).

The isolated yields were calculated after cleavage (44-58%, 12 examples), using two different selenylarylsulfonyl resins.

#### 2.1.3. Oxygen-based Linkers

Samarium (II) iodide is a useful reagent, exploited for several scopes, having the characteristic of being a powerful single-electron reducing agent. All the examples below, where fission of either a carbon-oxygen or a nitrogen-oxygen bond has been exploited to release organic molecules from a solid support, make use of samarium iodide.

##### 2.1.3.1. Cleavage of Hydroxylamine-based Linkers

**Scheme 36:**
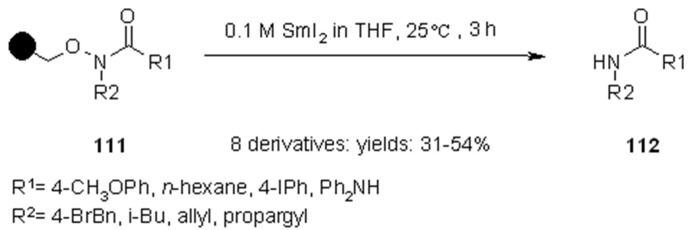
Abell’s amides and ureas library.

Abell and colleagues [40] used SmI_2_ for the traceless cleavage of *N-O* bond on substrates appropriately linked to SP. With this methodology it was generated a small library of amides and ureas (Scheme 36).

Although yields were modest, the purities of the cleaved products **112** were rather high.

**Scheme 37:**
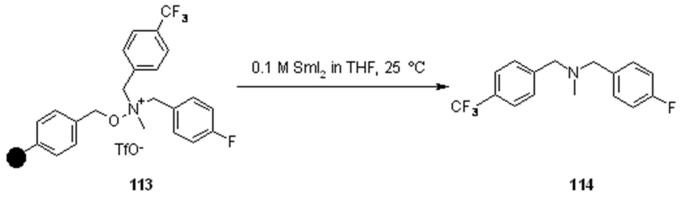
Andersson’s reductive cleavage.

The same cleavage technique was exploited by Andersson [41] that started from a polystyrene-based resin to synthesize tertiary amines (Scheme 37).

Although SmI_2_ gave clean cleaved products, the cleavage method used for the library production exploited the alternative LiI, which worked under neutral conditions.

**Scheme 38:**
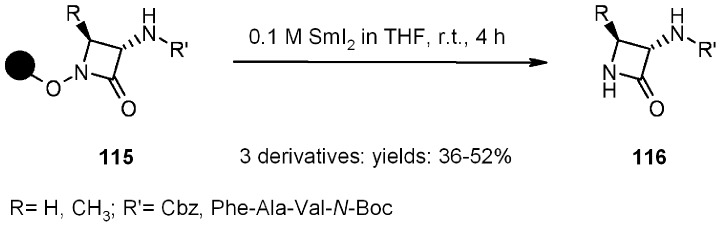
Taddei’s synthesis of β-lactams.

Another example of the reductive cleavage promoted by SmI_2_ was shown by Meloni and Taddei [42], who applied it to the synthesis of β-lactams (Scheme 38). Polystryerene-bound hydroxylamine was once again used as the starting material.

##### 2.1.3.2. Addition of Ketyl Radicals to α-β Unsaturated Esters

**Scheme 39:**
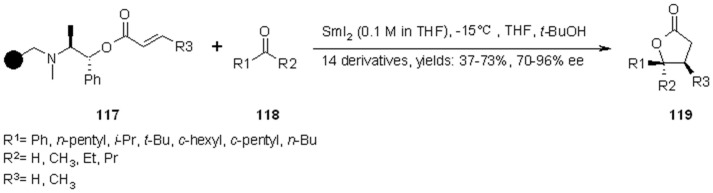
Procter’s asymmetric lactones synthesis.

Procter’s group [43,44] used a Sm(II) based radical chemistry for the enantioselective synthesis of γ-butyrolactones on SP exploiting (1*R*, 2*S*)-ephedrine linker as the chiral auxiliary. Bromo-Wang resin was first functionalized with (1*R*, 2*S*)-ephedrine and successively esterified at the alcohol group with the appropriate α,β-unsaturated acyl chloride to give **117**. This was, in turn, used in a reaction involving a ketyl radical addition to the anchored α,β-unsaturated ester. The subsequent cyclative cleavage led to the formation of lactone **119** (Scheme 39).

**Scheme 40:**
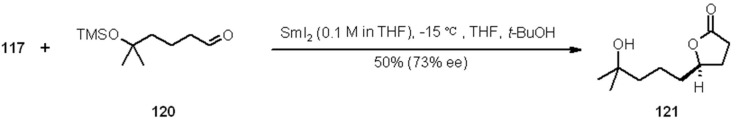
Synthesis of lactone **121**.

Under optimized conditions, the reaction gave rather high isolated yields (apart for a few exceptions) and diasteroselectivities. The same conditions were applied to the synthesis of lactone **121 **(Scheme 40), a moderate DNA-binding metabolite isolated from *Streptomyces* GT61115.

**Scheme 41:**
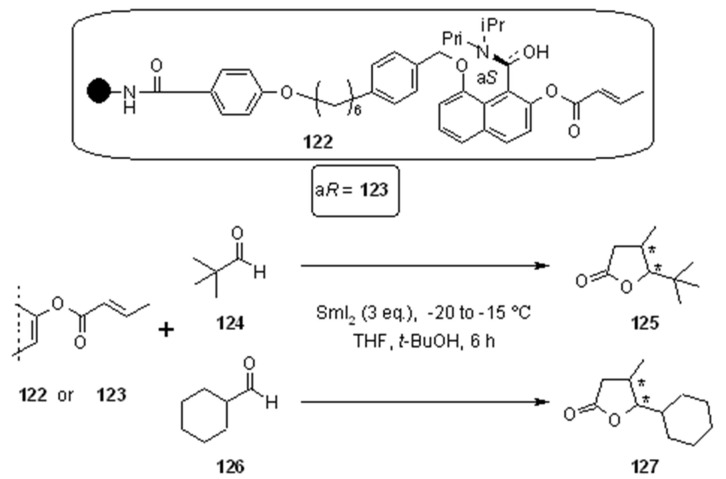
Dai’s chiral crotonates on Rink amide resin.

Cyclative cleavage gives back, beside the desired product, the resin functionalized with the asymmetric linker, which can be reused. Following Procter’s lead on asymmetric induction for samarium-mediated radical synthesis of γ-butyrolactones, Dai and coworkers [45] linked axially chiral crotonates on Rink amide resin for the same purpose (Scheme 41).

Reaction of **122** or **123** with aldehydes **124** and **126** gave reasonable isolated yields (**125** = 58%, **127** = 66%), with a good cis/trans selectivity (**125** = 71: 29; **127** = 89: 11) and good ee (**125**-cis: 94%, **125**-trans: 89%; **127**-cis = **127**-trans: 88%). The mechanism of chiral transfer was supposed to pass through an intermediate were the samarium atom coordinates the oxygens from **122** (or **123**) and the oxygen of the ketyl radical which performs the 1,4-addition to the crotonates.

##### 2.1.3.3. HASC Traceless Linker

**Scheme 42:**
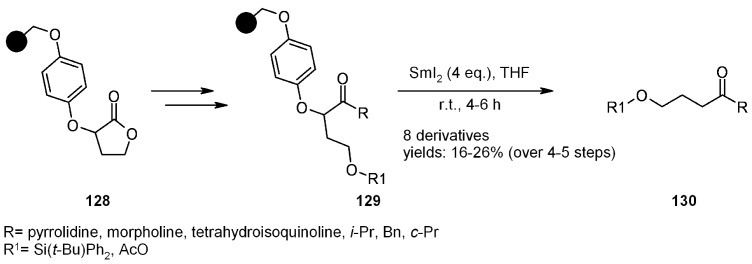
Procter’s application of HASC strategy.

The HASC traceless linker developed by Procter and mentioned above (see Scheme 8) has been exploited also using oxygen-based linkers. Applications of the ether linkage involve the synthesis of functionalized carbonyl compounds [46,47] (Scheme 42). Resin **128** was synthesized starting from bromo Wang resin, derivatized with the appropriate phenol and functionalised with α-bromo-γ-butyrolactone. Lactone ring-opening was performed with some amines (pyrrolidine, morpholine, tetrahydroisoquinoline and methoxymethylamine) and the resulting alcohol protected accordingly. When Weinreib hydroxylamine was used, the obtained tertiary amide was successively reacted with a Grignard reagent (BnMgCl, *i*-PrMgCl, *c*-PrMgCl) to yield the corresponding ketone. Cleavage of **129** from the resin gave products **130** in good yields after 4-5 steps.

#### 2.1.4. “Carbon–Based” Linkers

**Scheme 43:**
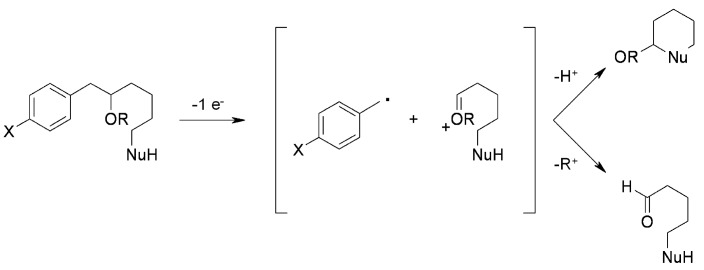
Floreancig’s proposed mechanism.

Floreancig’s group [48,49] used an oxidative release (often cyclorelease) on the appropriate polymer-supported precursors. The mechanism involved in the oxidative cleavage is presented in Scheme 43. 

**Scheme 44:**
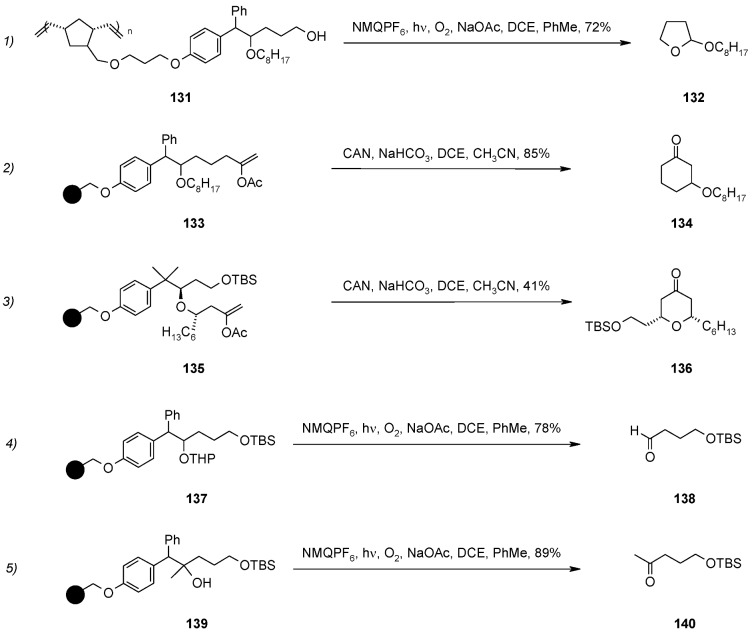
Application of lROMP polymers.

The process, (Electron Transfer Initiated Cyclization, ETIC) starts with single electron oxidation and mesolytic cleavage of the *C-C* bond of the homobenzylic ether, forming a benzylic radical and an oxocarbenium ion. The resulting cation is successively transformed in a neutral species, giving a cyclised product if an appropriate nucleophile is present in the molecule, or an aldehyde (or ketone) if there is loss of the oxygen-bound “R” group (when present). To test this methodology on support, appropriate soluble ROMP polymers were prepared (Scheme 44).

The five equations prove the feasibility of the method, either by intramolecular trapping (equations 1-3), or by oxidative traceless release (equations 4-5). In the former case either formation of a *C-O* bond (equation 1) or a *C-C* bond (equation 2 and 3), which occurred with high diasteroselectivity, is shown. In the latter case formation of the aldehyde **138** of equation 4 requires loss of a THP group.

The purity of the products after this particular cleavage was rather high, due to the characteristic mechanism of the reaction, which ensured cyclocleavage or oxidative release only when the appropriate nucleophile was correctly installed in the molecule. This means that any impurity will be retained on the support, in a kind of purification step. Therefore, it is possible to perform several steps on the support, without the fear of accumulating side-products that have to be separated at the end of the process. 

**Scheme 45:**
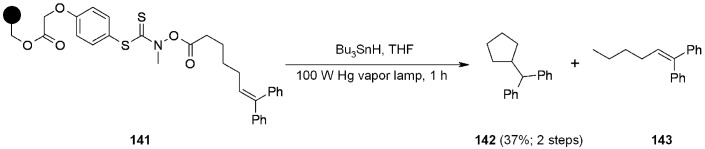
Routledge’s THA linkeras polymer-supported radical source.

An interesting application of thiohydroxamate (THA) linker strategy was proposed by Routledge [50]. In previous reports [51,52] the same group presented a traceless THA linker, cleavable under photolytic conditions, but in the need of novel SP-linkers, a second-generation THA linker, stable under diverse chemical and photolytic conditions, was studied (Scheme 45).

Although **141** proved to be stable to heat and light, it was sensitive to nucleophilic cleavage, which might make it of limited application for SP preparation of libraries. On the other hand, **141** was found useful as polymer-supported radical source. The nature of the linker allows the generation of a carbon-centred radical ultimately arising from a carbon-carbon bond fission, that can be appropriately exploited for successive reaction such as the one depicted above. The only product found was **142**, even if isolated in moderate yield.

### 2.2. Solid-Supported Reagents

Another important aspect of radicals exploiting SP deals with the use of supported reagents for radical reactions. This combination allows toxic reagents, often difficult to separate from the desired products in solution, to be used with ease of purification and little contamination. Since the most important reagents in radical chemistry are represented by organostannanes, it was obvious that the initial efforts to translate in-solution radical chemistry to SP, would have involved tin-base reagents.

**Scheme 46:**
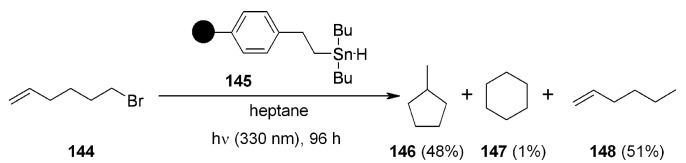
Use of resin **145** for radical cyclization.

From this point of view, several works have tried to bind organostannanes on solid supports, in order to reproduce common radical reactions and make work-up easier with little or no contamination from the toxic metal. Within this class, organostannylhydrides occupy a dominant role. Neumann’s group successfully immobilized dibutyltinhydride on SP with a two-carbon spacer between the metal and the aromatic ring of polystyrene to obtain resin **145**, whose applications were highlighted in successive publications [53,54]. When employed for radical cyclization (Scheme 46), the results obtained were comparable with the same reaction run in solution with Bu_3_SnH (36%:0%:64%).

**Scheme 47:**
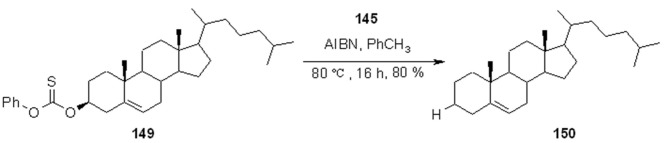
Barton dehydroxylation using resin **145**.

**Scheme 48:**
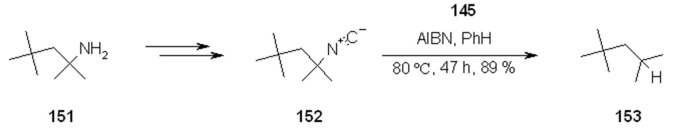
Deamination reaction using resin **145**.

An application to the Barton dehydroxylation reaction [55], which has been exploited extensively later by the same group in a series of derivatized alcohols [56], is shown in Scheme 47. An example of deamination following preparation of the correspondent isocyanides is instead shown in Scheme 48.

The supported tin hydride was recycled by reduction of the filtered resin, maintaining a good efficiency. Following a similar path, Dumartin and coworkers [57,58] synthesized supported organostannanes with spacer of 2, 3 and 4 carbon atoms, in order to avoid β-elimination of the stannane as by-product, therefore producing recyclable and more stable reagents for radical reactions. In a successive paper [59], the same group used the anchored –Bu_2_SnX (X = halogen) with a C-4 spacer for catalytic reductions of haloalkanes in the presence of NaBH_4_ and AIBN. The length of the spacer proved to be important for reaction yields (due to more accessibility of the metal centre) and residual pollution by the metal.

Deleuze [60] prepared a macroporous organotin chloride by inclusion of a suitable organostannyl-styrene precursor in the polymerization reaction. The so prepared supported reagent (in combination with NaBH_4_) proved to be comparable to Bu_3_SnH in reducing alkylhalides in solution.

Dumartin’s group [61] investigated the possibility to use tin-based reducing agent in catalytic amount, as it had been done in solution [62]. In particular, their attention focused on the use of siloxanes, particularly polymethylhydrosiloxane (PMHS), for the regeneration of catalytic amounts of TBTH. However, PMHS proved ineffective using polymer-bound organostannane **154**, evidently because it was incapable of penetrating the PS beads.

**Scheme 49:**
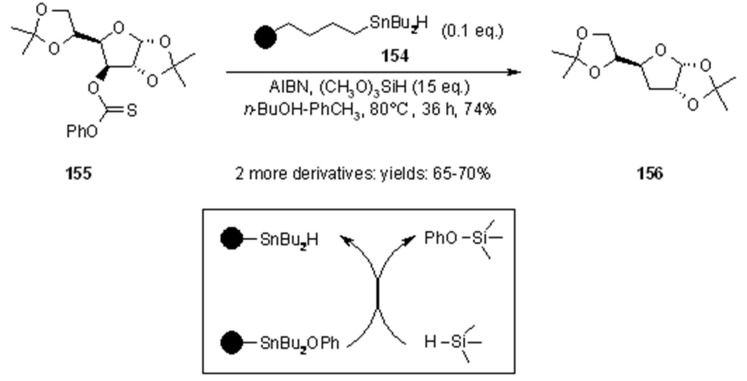
Dumartin’s catalytic approach.

**Scheme 50:**
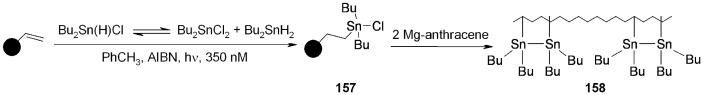
Neumann’s organodistannane.

An alternative was chosen and found in trimethoxysilane, giving good results. The reactivation of the tin by-product is highlighted in the scheme (Scheme 49). An alternative to organostannyl hydrides in radical chemistry is represented by the combination organodistannanes/sensitizer/H-donor *via*, for example *hυ* initiation. Neumann also studied anchored distannanes on SP [63,64], which can easily be prepared starting from the commercial polystyrene. The tin chloride derivative **157** was synthesized by conventional chemistry, and subsequently treated with Bogdanivic’s magnesium (Mg-anthracene-3THF) to form the distannane **158**.Scheme 50

This could be used as the equivalent non-supported reagent in the addition of *tert*-butyl iodide to acetylenes, yielding similar results. However, the advantage is that after the reaction the polymer containing SnI groups can be recovered by filtration, and completely regenerated in a straightforward manner by reaction with Bogdanivic’s magnesium.

**Scheme 51:**
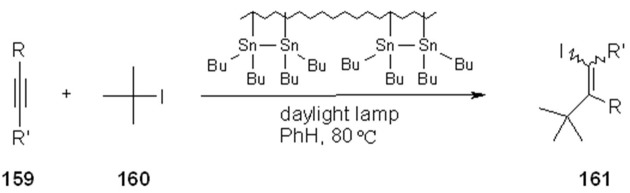
Addition of *tert*-butyl iodide to acetylenes.

**Scheme 52:**
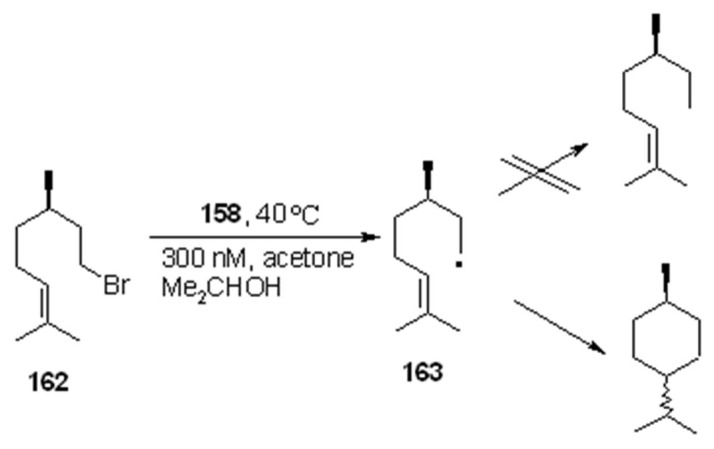
Resin **158**-mediated radical cyclization.

Interestingly, when the polymer was treated with the bromide **162** in the presence of acetone as long-lived triplet sensitizer, and 2-propanol as weak *H*-donor, the corresponding radical **163** thus generated evolved in a somewhat unexpected manner. In fact, instead of the anticipated reduction, a cyclization product was obtained.Scheme 52

Generally speaking, the possibility to chose the *H*-donor strength (and, therefore, to direct the radical reaction in one of the competitive pathways), the ease of separation of the tin-residues and the little contamination, make this option reagent a feasible suitable choice.

**Scheme 53:**
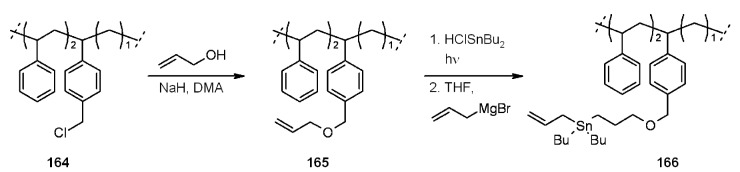
Enholm’s allylstannane.

Enholm and colleagues [65] studied the use of allystannane anchored to SP. This toxic reagent is used in the allylation of organohalides (or other homolytic functions) under radical conditions and suffers of the common problems encountered with tin-containing reagents. The use of supported tin reagent leads to ease of purification and minimal contamination of the products. In order to enhance the rates of the intermolecular reaction at test, it was chosen a soluble non-cross-linked polystyrene support (Scheme 53), which induces a 100-fold increase in the rate of intermolecular radical reaction compared to the heterogeneous classical SP reactions. 

**Scheme 54:**
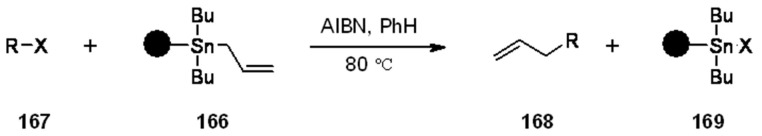
Application of allylstannane **166**.

It was found that the best combination <number of reactive sites-polymer properties> led to a 33% of loading capacity. Test reaction is shown in Scheme 54.

**Scheme 55:**
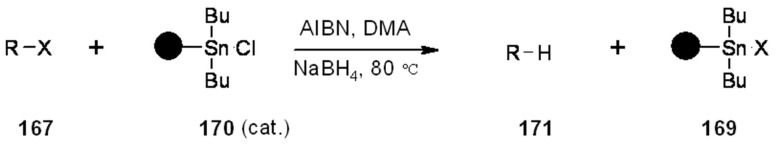
Enholm’s reduction of organohalides.

Without going through the details, it is worth underlining some features of the methodology: electron-deficient substrates worked best, while electron-rich did not work at all. This behavior was attributed to the “philicity” of the generated tin radical, which is rather electron-rich (nucleophilic), generating a mismatch with the radical substrate. Interestingly, tertiary radicals, that gave no reaction in solution, gave a 50% yield. Finally, this procedure displayed a strong regioselectivity towards dihalides substrates, yielding a product of single allylation, strongly preferring electron-deficient radicals. In an extension of the precedent methodology, the same group [66] performed the preparation of another organotin-supported reagent for the reduction of organohalides (Scheme 55).

The preparation of **170** was performed following the synthesis depicted in Scheme 53, where **165** was only treated with HClSnBu_2_. The catalytic methodology used by Enholm is well established [67]. It is widely used for radical reduction of organohalides and represents a useful way to avoid the excess of tin-derivatives, toxic and difficult to separate. The application of the catalytic strategy to the SP allows a low loading of tin-supported reagent and, in addition, the use of a soluble support speeds up the reaction times compared to heterogeneous analogue reactions. Primary alkyl halides reacted quickly with 0.1 eq. of **170** and when the amount of supported reagent was lowered, some reactions took longer. It is worth noting that use of a dihalides with only 1 equivalent of **170** gave only one product of reduction.

Contamination with tin was evaluated *via* degradation of the products followed by inductively coupled plasma mass spectrometry (ICP-MS), showing the presence of very low residual amount of the metal (around 10 ppm).

**Scheme 56:**
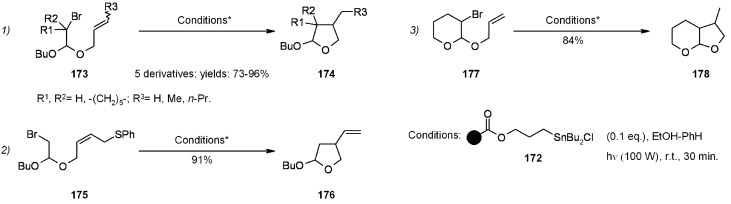
Ueno’s synthesis of 2-alkoxytetrahydrofurans.

In a very early example of solid-supported organotin reagents, Ueno and coworkers [68] compared in-solution radical cyclizations under classical conditions (Bu_3_SnH/AIBN) with the use of polymer-bound radical carrier (Bu_3_SnCl/NaBH_4_) for the synthesis of 2-alkoxytetrahydrofurans (Scheme 56).

It was found that the reactions were higher-yielding when polystyrene-bound organotin chloride **172** was used as radical carrier. The supported reagent could be used several times after appropriate washing.

Trialkylgermanium hydrides have shown a high potential in radical chemistry often hindered by their high costs, due to their lower toxicity in comparison to that of the corresponding organostannane.

In the need of new reagents, Mochida’s group [69] studied the synthesis of polymer-supported germanium hydrides, either *via* functionalization of polystyrene or by addition to the polymerization mixture of a suitable germanium-derivatized styrene. The supported reagents were tested for the reduction of organic halides, finding comparable results to known in-solution analogous reactions. In addition, it was demonstrated the opportunity of regeneration of the insoluble reagent by reduction with LiAlH_4_.

**Scheme 57:**
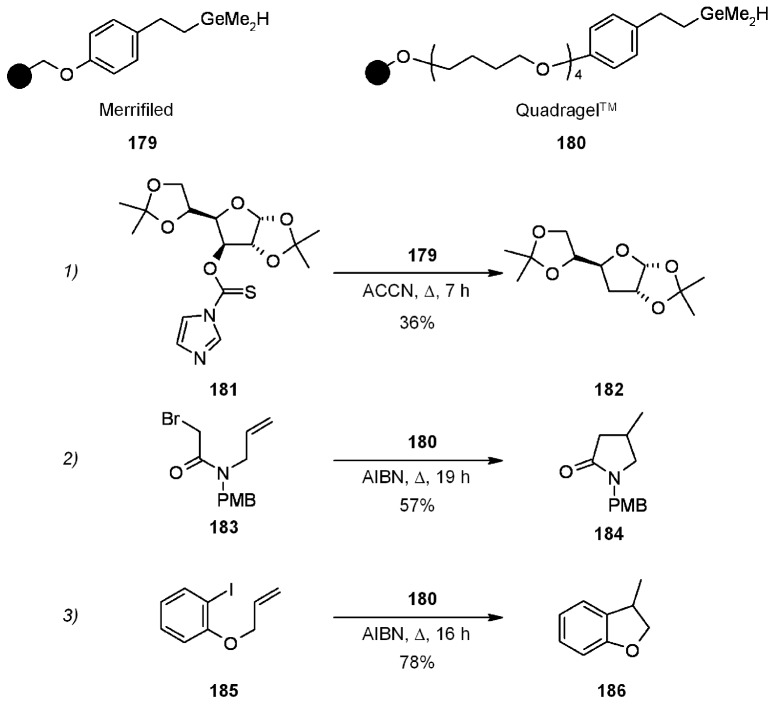
Bowman’s use of SP- germanium hydrides.

Bowman’s group [70] also performed the synthesis of resin-bound germanium hydrides. Examples of their applications, which always gave reasonable results, are shown below (Scheme 57).

**Scheme 58:**
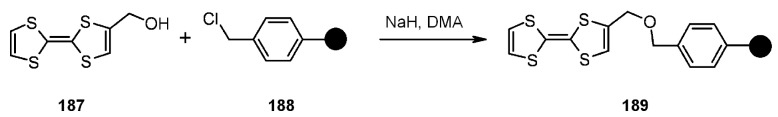
Murphy’s synthesis of PS-TTF.

In the quest of radical sources on SP, the work presented by Murphy’s group [71] represents an alternative to the immobilized organostannanes previously described. Tetrathiofulvalene (TTF) is the promoter for the radical-polar crossover reaction [72,73,74], a methodology used to generate aryl radicals from aryldiazonium salts. The reaction run in solution suffers from the need for separation of TTF by-products and Murphy’s goal was immobilization of the promoter in order to simplify work-up (Scheme 58).

**Scheme 59:**
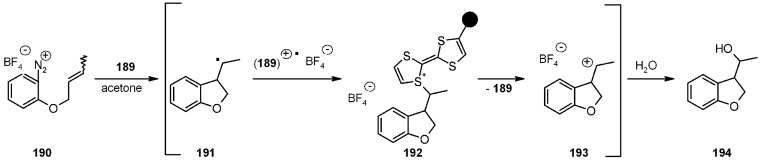
Murphy’s use of PS-TTF.

Hydroxymethyltetrathiofulvalene **187** was prepared and loaded to a gel-type resin and used for test in radical cyclization reactions, as the example shown below (Scheme 59).

The reactions tested were successful, with comparable results with the homologous run in solution, even if slightly lower-yielding. A major concern was the presence of side-products such as the deoxygenated analogue of **194**, possibly arising from H-abstraction by the radical intermediate **191** from the polymer benzylic hydrogens, or products of reaction of cation **193** with the aryl backbone of the resin. In both cases, it was proved that none of these concerns was factual and the reactions resulted rather clean. In addition, contamination of the products from TTF by-products was absent and it was also proven that the SP-TTF could be recycled after regeneration by reduction with NaBH_4_ in methanol.

**Scheme 60:**
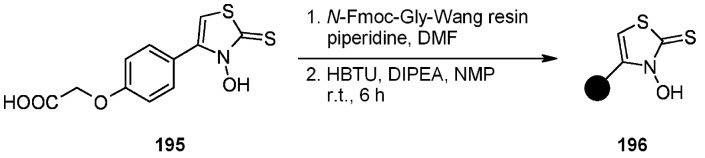
Giacomelli’s interpretation of Barton-Crich thiohydroxamates chemistry.

Giacomelli and colleagues [75] successfully attempted a useful extrapolation of the classic Barton-Crich thiohydroxamates chemistry [76] in a work where a new polymer-supported radical source was prepared and tested. *N*-hydroxythiazole 2(3)-thione **195** was synthesized and loaded on a Gly-Wang resin to obtain radical source **196** (Scheme 60).

**Scheme 61:**
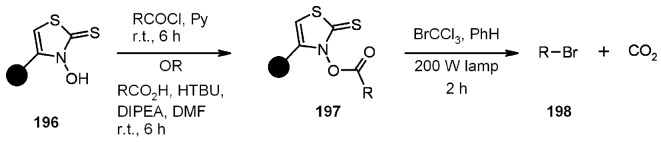
Use of resin **196** for the generation of alkyl radicals.

**Scheme 62:**
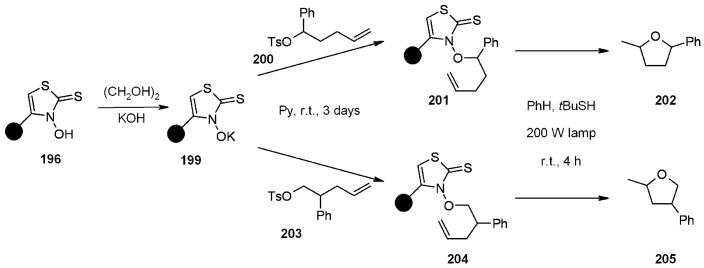
Use of resin **196** for the generation of alkoxyl radicals.

The polymer-supported reagent was then tested for the generation of alkyl radicals (Scheme 61), where R = C(CH_3_)_3_; CH_2_Ph; CH(Me)CH_2_Ph; CH_2_CH_2_CH(NHBoc)COO*t*-Bu) and alkoxyl radical (Scheme 62).

In both cases the reactions were successful, proving this methodology valid for the generation of radicals and having the additional advantage of ease of purification compared to the often tedious experience of purifying the in-solution analogues.

**Scheme 63:**
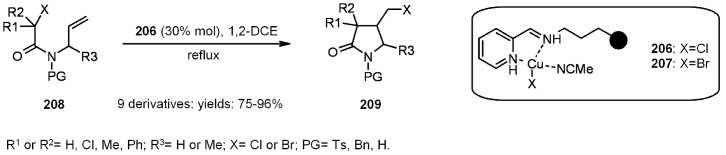
Clark’s ATRC approach.

Transition-metal-catalyzed atom transfer radical cyclization (ATRC) represents an alternative, tin-free, non-reductive, catalytic, radical cyclization methodology. Clark’s group [77,78] studied the efficiency of this method for the construction of small molecule by anchoring the ligand to SP. In a first attempt [77], functionalized silica was used for the support of the *N*-alkyl-2-pyridylmethanimine ligands (Scheme 63).

**Scheme 64:**
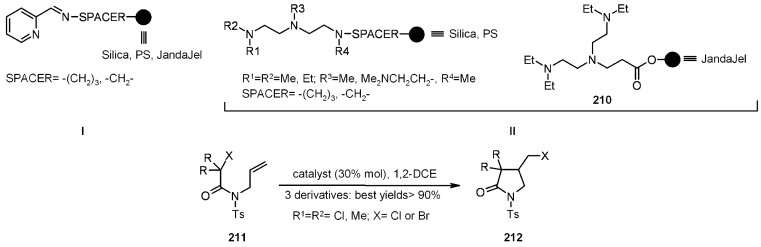
Clark’s catalyst improvements.

All the reactions were high-yielding, the major difference among them being the time necessary to get to completion (from 1 to 36 h). The stereochemistry is not shown, although the *trans* isomer resulted to generally be the major one. The catalysts **206** and **207** were prepared by treatment of the immobilized ligand with CuX and **206** was also reused at least 3 times, giving still good yields (92→90→86%), but longer reaction times (3→24→36 h), a deactivation probably caused by the formation of an inactive CuCl_2_ complex. In a successive paper, Clark [78] used different supports and several ligands, in order to determine the best combination to improve the efficiency of the reaction (Scheme 64).

**Scheme 65:**
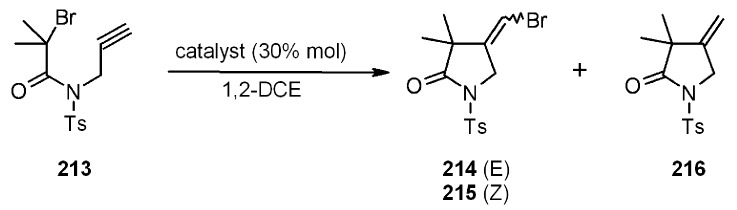
Example of radical addition to a terminal alkyne.

Running the above reaction at room temperature, some differences among the catalysts (obtained after treatment of supported ligands of type **I** or **II** with CuX) arose, since there was a clear superiority of those of type **I** *vs.* type **II**, in terms of reaction times and conversions (yields were, however, rather high). Raising the temperature to reflux, the reaction times were less than 30 min, independently of the catalyst used. Some more substrates were investigated, giving interesting results. For instance, in (Scheme 65) the catalyzed radical addition to a terminal alkyne is described. The absolute yields were high (88-98%), independently of the ligand used, even if type **I** catalysts were slightly better. *E:Z* ratio was almost the same for the ligand screening, however, the presence of reduced product **216** varied between 11-6%, being 0% when silica type **I** was used.

**Scheme 66:**
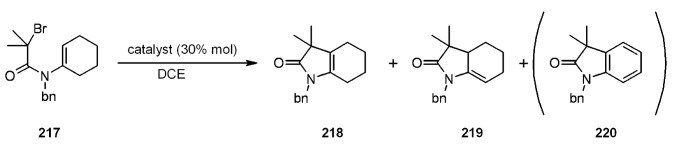
Clark’s 5-*endo*-trig radical polar crossover addition.

In Scheme 66 is depicted a 5-*endo*-trig radical polar crossover addition, a normally disfavored process following Baldwin’s rules [79,80], but feasible when polyamine-copper catalysts such as type **II** (Scheme 64) are used [81]. In this case a mixture of **218** and **219 **was obtained. The best yield (100%) was found when **210** was used as ligand, while **220** was isolated in low yield (15%) as the sole product when type **I** Janda Jel ligand was used.

**Scheme 67:**
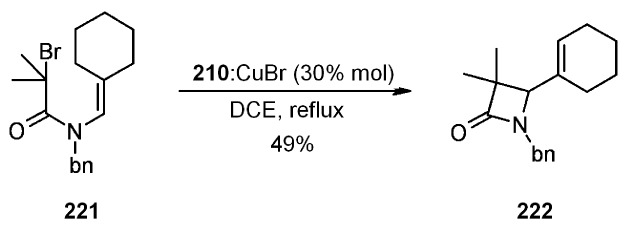
Clark’s 4-*exo*-trig radical cyclization.

Finally, in Scheme 67 a 4-*exo*-trig cyclization is represented. This also is a disfavored process according to Baldwin’s rules, which was successful only when **210** was used as ligand.

**Scheme 68:**
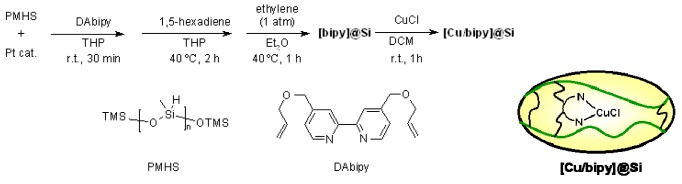
Nagashima’s Cu-catalytic complex.

Nagashima and coworkers [82] studied the same reaction, using different copper complex as the catalyst. In this case, in fact, the metal-ligand complex used for the catalysis was immobilized on a very peculiar SP the (PMHS). The siloxane gel [bipy]@Si contained, embedded, a bypyridine ligand and it was synthesized from PHMS and 4,4′-bis(allyloxymethyl*)-*2,2′-bipyridine (DAbipy) by the platinum-catalyzed hydrosilylation of alkenes. The catalyst was then tested on several radical precursors (Scheme 68).

The synthesis of the actual catalyst [Cu/bipy]@Si was completed after treatment of [bipy]@Si with CuCl. The metal was easily soaked into the gel, forming the active complex, which resulted more stable to air than the not-embedded version and that can be filtrated from the organic solvents used for the reaction and re-used with small loss of TON. Metal leakage inferior to the detection limit was observed.

**Scheme 69:**
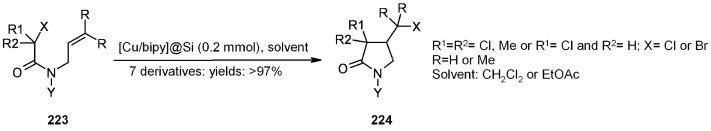
Application of [Cu/bipy]@Si to 5-*exo*-trig radical cyclization.

**Scheme 70:**
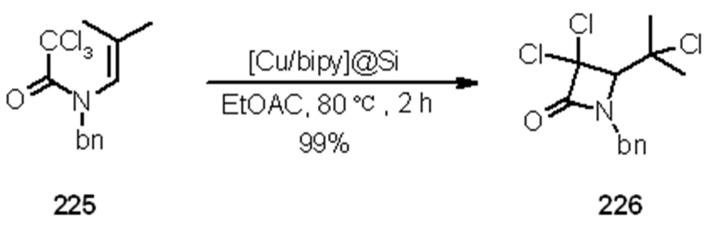
Application of [Cu/bipy]@Si to 4-*exo*-trig radical cyclization.

The reactions shown in Scheme 69 and Scheme 70 were all pretty successful (97-99%) and when the reaction gave low yields at room temperature, simple heating gave almost complete conversions. 

**Scheme 71:**
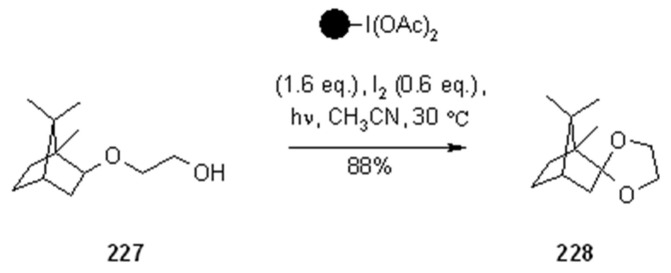
Togo’s synthesis of 1,3-dioxolanes.

**Scheme 72:**
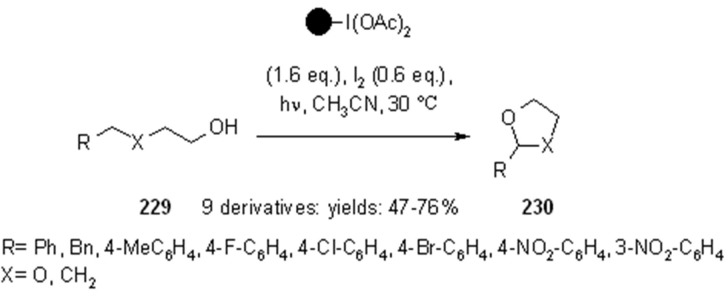
Togo’s synthesis of 2-substituted tetrahydrofurans and dioxolanes.

**Scheme 73:**
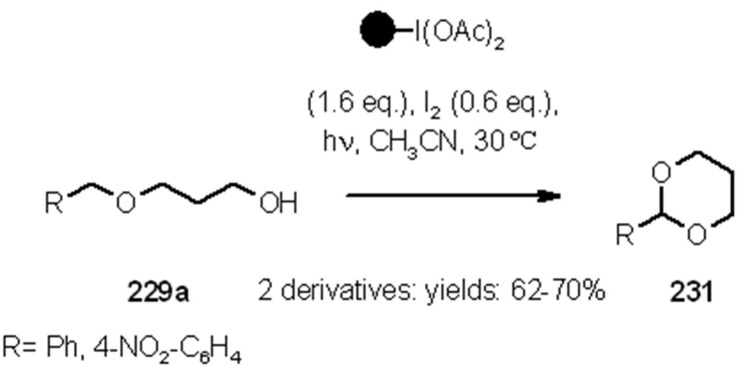
Togo’s synthesis of 2-substituted-1,3-dioxanes.

Hypervalent iodine reagents can be used for the generation of alkoxy radicals under irradiation conditions. Teduka and Togo [83] used polystyrene-supported (diacetoxyiodo)benzene for the synthesis of 2-substituted 1,3-dioxolanes (Scheme 71), 2-substituted tetrahydrofurans (Scheme 72) and 2-substituted 1,3-dioxanes (Scheme 73).

**Scheme 74:**
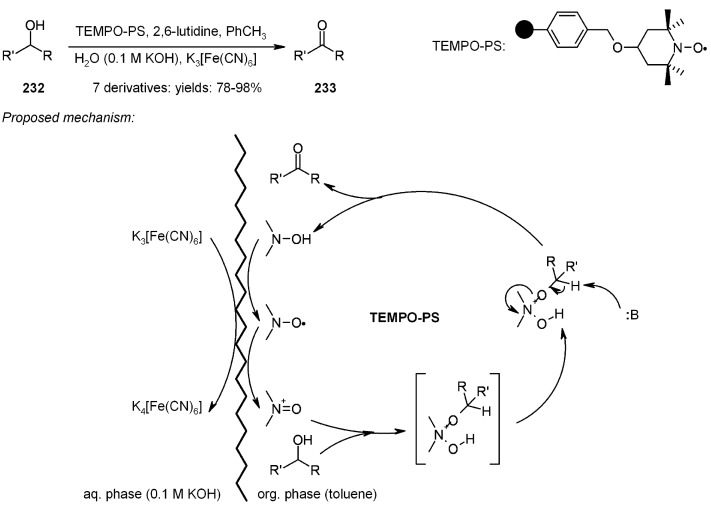
Kashiwagi’s catalytic two-phase system.

Oxidation of alcohols to the corresponding aldehyde or ketone represents a very common reaction in daily organic synthesis; however, there is always a need for new and milder methods for sensitive molecules, especially in SP organic chemistry. Kashiwagi’s group [84] studied a mild and efficient methodology, using a catalytic two-phase system, under basic conditions. (Scheme 74)

The oxidation mechanism is displayed above and consists of an interface interaction between the oxidant (K_3_[Fe(CN)_6_]) in aqueous, basic solution and the hydroxylamine polymer-bound (TEMPO-PS) in organic phase (toluene), formed after oxidation of the alcohol **232**. The base (2,6-lutidine) was necessary to close the catalytic cycle, yielding the desired carbonyl **233**. Interestingly, in was found that this method has a rather high chemoselectivity, preferring primary alcohols oxidation to secondary alcohols. 

## 3. Exploitation of Radical Chemistry in SP Synthesis

The importance of radicals in organic chemistry has grown considerably over the past three decades and several methodologies have been studied and set up to face the need of new routes for the synthesis of target molecules or classes of compounds. In this section, the studies and applications of those methodologies to SP will be discussed. Following our goal to provide a reference for the day-to-day use of solid-phase radical reactions, we have not based our classification on mechanistic grounds, but more simply on the formal way to generate a new bond. 

### 3.1. Carbon-Carbon Bond Formation through Intermolecular Reactions

#### 3.1.1. Substitutions Alpha to Carbonyl Groups

**Scheme 75:**
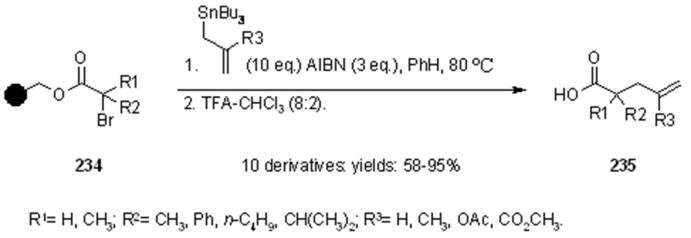
Sibi’s allylation reaction on supported α-bromo.

The first examples of intermolecular free radical allylation reaction on solid support was reported by Sibi and Chandramouli [85], for the synthesis of simple α-allyl acetic acids (Scheme 75).

**Scheme 76:**
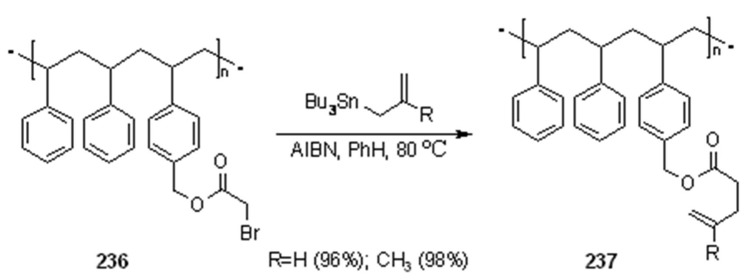
Enholm’s allyl transfer reaction.

Wang resin was used as the solid support and the reactions were carried out using allyltin derivatives. As shown in the scheme, the reactions were rather successful and allylation was more effective (95% yield) when R^3^ was either and electron-donating (OAc) or electron-withdrawing (CO_2_CH_3_) group, while substitution around the C-centered radical gave lower yield (58%) for R^1^ = R^2 ^= CH_3_. Allyl transfer reaction was studied also by Enholm’s group [86] who worked on a soluble support (Scheme 76).

The polymer (non-cross-linked polystyrene) was functionalized to obtain the radical precursors. As shown in Scheme 76, **236** could be reacted with allyltributyltin derivatives under radical conditions, allowing allylation of the carbonyl α-carbon.

**Scheme 77:**
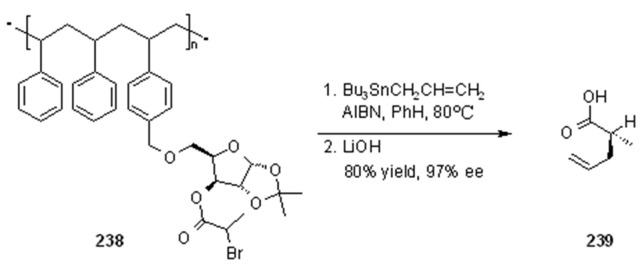
Enholm’s allyl transfer reaction.

Allylation of the same radical precursor (α-bromo carboxylate) linked this time to the polymer by means of a sugar moiety is shown in Scheme 77. When the reaction was performed in solution, use of a Lewis acid increased stereoselectivity. However, in the case shown in Scheme 77 any Lewis acid (LA) used gave premature cleavage. Fortunately, in this case a good stereoselectivity (97% ee) was found without any LA addition: this can be explained invoking the Lewis acidity of Bu_3_SnBr (formed during the reaction), which coordinates preferentially to one face of the sugar molecule directing the allyl attack. Enholm studied a series of intermolecular radical reactions also exploiting ROMP polymers [87] (see below).

**Scheme 78:**
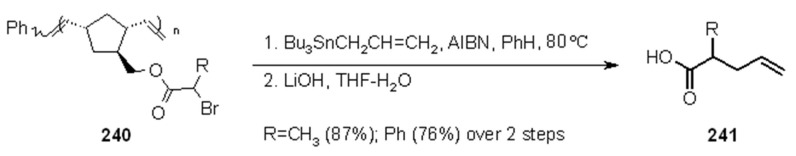
Enholm’s use of ROMP polymers for the synthesis of **241**.

**Scheme 79:**
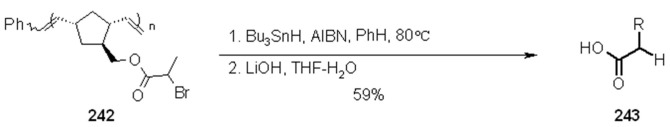
Enholm’s use of ROMP polymers for the synthesis of **243**.

**Scheme 80:**
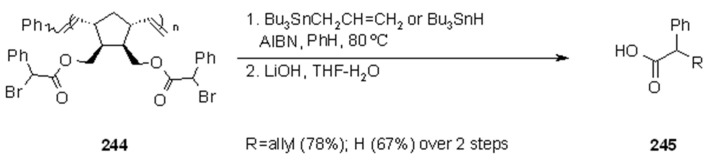
Enholm’s use of ROMP polymers for the synthesis of **245**.

The polymers **240, 242 and 244** (Scheme 78, Scheme 79, Scheme 80) were prepared by building the appropriate norbornene precursors that were subsequently treated with Grubbs’ catalyst (see below). Treatment of the so obtained soluble polymers under radical conditions gave the desired products of allylation (Scheme 78) or reduction (Scheme 79) in good yields. A tremendous advantage of this type of polymers is represented by the presence of at least one molecule of substrate in each monomer, giving a 100% loading capacity to the polymer. In the case of Scheme 80, where each monomer contains two reactive sites, a 200% theoretical loading of the resin was obtained.

**Scheme 81:**
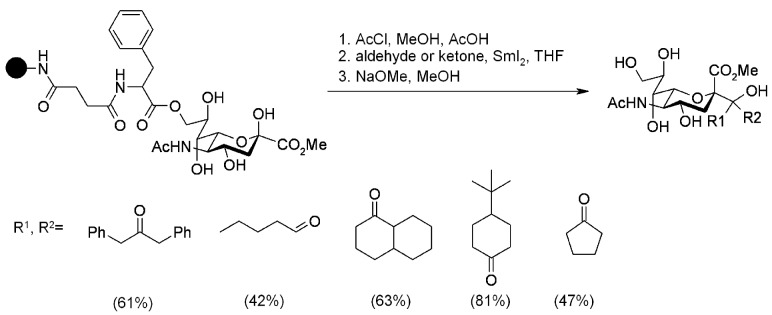
Linhardt’s *C*-glycosilation.

Linhardt and coworkers [88] used SmI_2_ as a SET reagent for the *C*-glycosilation of Neu5Ac on SP. Since carboxyl TentaGel resin failed to give any desired product, the reaction was run on a less hydrophilic material, namely a functionalized controlled pore glass (CPG). The glass was derivatized in order to present an amine group handle (Scheme 81). Methyl ester of neuraminic acid was first esterified with a spacer that was subsequently attached to the support. As shown in the scheme below, the results of the *C*-glycosilation reaction were rather satisfactory.

#### 3.1.2. Radical Addition to Alkenes

**Scheme 82:**
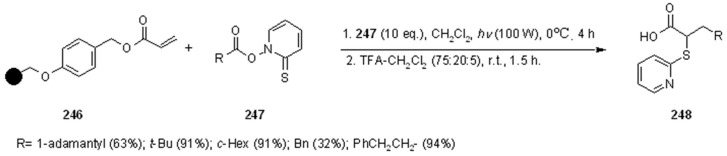
Ganesan’s radical addition to supported alkenes.

Radical addition to unsaturated *C-C* bonds represents one of the most common reactions in radical chemistry. The efficacy of the intermolecular versions of such reactions is heavily dependent on the nature of reaction itself. An early example of this chemistry on SP was presented by Zhu and Ganesan [89], that proved the efficiency of alkyl radical addition to acrylates attached to a chosen resin (Scheme 82).

**Scheme 83:**
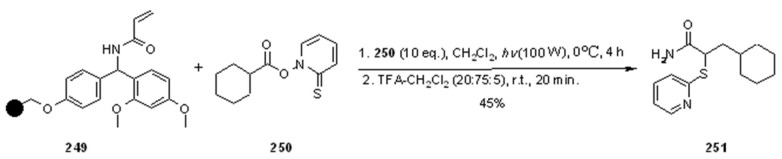
Ganesan’s radical addition using Rink resin.

The alkyl radicals were generated by photolysis of the corresponding Barton esters **247** and addition was generally good, with the exception of the stabilized benzyl radical that gave poor conversion (yields refer to the use of PS-Wang resin, as Tentagel-Wang gave lower yields). The reaction was performed using a Wang (Scheme 82) or a Rink (Scheme 83) resin. In the latter case yield was lower, a result in accord to the in-solution reaction, probably due to the differences between acrylate ester **246** and acryl amide **249**. 

**Scheme 84:**
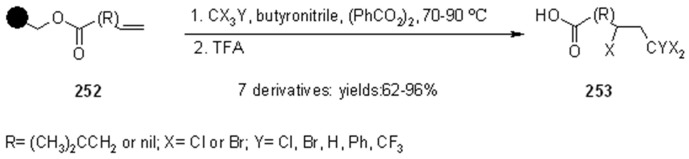
Kumar’s radical addition to supported alkenes.

1,2-Radical initiated addition of haloalkanes to *C=C* bonds is employed in commercial routes for the preparation of dihaloethenylcyclopropane carboxylic acids, and the development of cleaner and easier methods has brought Kumar and colleagues [90] to attempt the reaction anchoring the olefin to SP (Scheme 84).

**Scheme 85:**
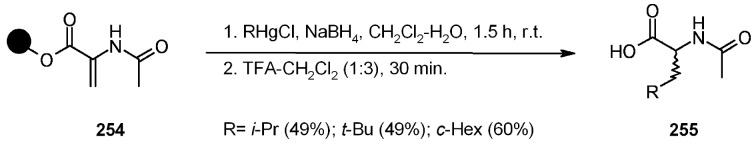
Yim’s radical addition to supported alkene.

The olefins were linked to Wang or Merrifiled resins by their carboxylic unit and the reactions were run in the presence of butyronitrile as a cosolvent (except when CCl_4_ was used as reagent). Yields varied from moderate to high, for reaction times between 24 and 48 h, making this method effective, but rather slow. The synthesis of racemic α-amino acids was described by Yim [91] that applied the mercury method for the generation of alkyl radicals in the presence of polymer-supported dehydroalanine (Scheme 85).

Addition of the alkyl radical on the double bond was effective and after cleavage reasonable yields of the desired products were isolated. When the same reaction was performed under more usual radical conditions (Bu_3_SnH, AIBN, *t*-BuI, PhH, under reflux), only trace amounts of the product (8%) were isolated, while prolonged heating led only to decomposition. The reason for this behavior was attributed to the poor swelling of the Wang resin in the solvent used in the attempt (benzene). 

**Scheme 86:**
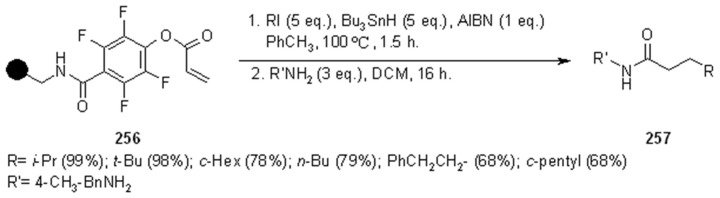
Caddick’s radical addition to supported alkene.

Caddick’s group [92] prepared a small array of amide derivatives by intermolecular radical addition of C-centered radicals to an activated acrylate anchored to a resin. Aminomethyl-polystyrene resin was chosen as the SP where to anchor the acrylate ester of 2,3,5,6-tetrafluoro-4-hydroxybenzoic acid (**256**). The choice of such electron-poor acrylate was made in order to facilitate the radical reaction and obtain an amide after cleavage with an appropriate amine. The general synthetic scheme of this high-yielding methodology is represented in Scheme 86.

**Scheme 87:**
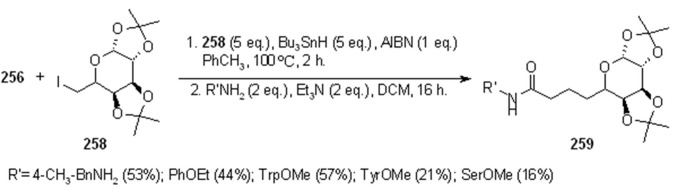
Synthesis of compound **259**.

**Scheme 88:**
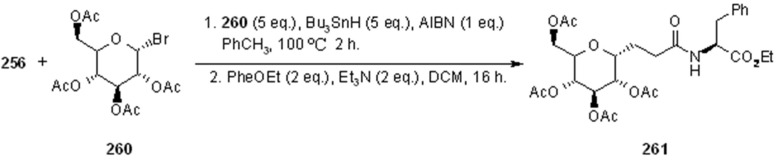
Synthesis of compound **261**.

In Scheme 87 and Scheme 88 the use of carbohydrate derivatives as radical precursors (**258** and **260** respectively) is shown. In these cases, α-amino acids were used as amines, although the yields were lower compared to the previous case.

#### 3.1.3. Radical Addition to Oxime

**Scheme 89:**
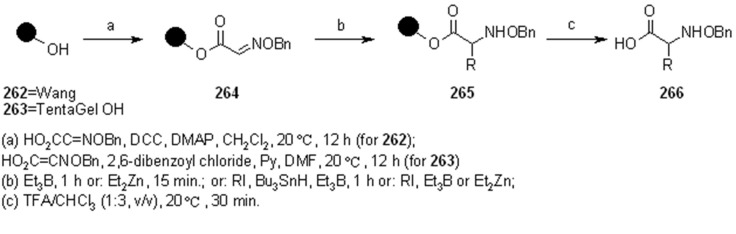
Naito’s radical addition to supported oxime.

Among the literature references regarding the use of radical chemistry on SP, Naito’s group contributed by studying the reactivity of SP-supported oxime ethers. In their works [93,94,95,96,97], they studied the addition of appropriate alkyl iodides to polymer-supported glyoxylic oxime ether, giving the first example of intermolecular radical reaction exploiting triethyl borane or diethyl zinc as radical initiators. The methodology was used for the synthesis of unnatural aminoacids (Scheme 89).

After loading the oxime to the solid phase (**264**), a first attempt was made using only Et_3_B in DCM, which worked as initiator and terminator to afford, after cleavage, aminoacid **266** where R = Et. Running the reaction with an alkyl iodide R-I in the presence of Bu_3_SnH, a range of products were obtained, thus, when R = *i*-Pr, *c*-hexyl, *t*-Bu, *s*-Bu, the corresponding unnatural amino acids were recovered in good yields, while the unstable primary radical *i*-Bu and the bulky adamantyl gave lower yields. In all the reactions, a small amount (5-30%) of **266** (R = Et) was found as by-product from the competitive addition of ethyl radical arising from Et_3_B. The reaction was also run in the presence of Et_2_Zn, which gave comparable results to the Et_3_B; in both cases the radical chain was effective also at low temperatures (-78 °C). Successively, they tried to run the reaction under iodine-atom transfer mechanism, using Et_3_B and *i*-PrI, but avoiding Bu_3_SnH. In this case a large amount of product where R = Et was found. The increased competition of the side-reaction was not explained, however, a change in solvent and temperature (toluene, 80 °C) as well as the use of a larger amount or alkyl iodide (60 eq.) gave a better selectivity towards the desired product (5.7:1 *vs.* 2:1). Such selectivity, however, remained lower compared to the reaction run in solution.

In general, when the reaction was run in the presence of an organic solvent, Wang resin proved to be slightly better compared to TentaGel OH. However, when the reaction was tried in aqueous media, which is normally effective in solution, Wang resin did not work at all because of its poor swelling properties. Success was achieved with TentaGel OH resin, where it was necessary to use a solution of Et_3_B in THF or MeOH (compared to the hexane solution used in the previous attempts) to obtain a monophasic solution. The reaction was run in a mixture H_2_O-MeOH (2:1, v/v, at 70 °C), under iodine atom transfer conditions and the overall yields were acceptable though lower compared to the ones run in organic solvents; a comparable amount of ethylated product was obtained.

**Scheme 90:**
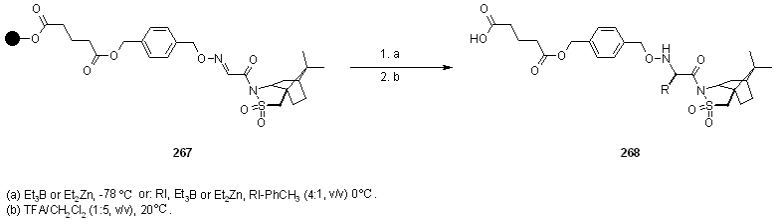
Naito’s stereoselective radical addition to supported oxime.

Although the methodology described by Naito was biased by the presence of the undesired product of ethyl radical addition, the use of aqueous solvents and the absence of the toxic tin hydride make this procedure quite appealing. The same group also studied the stereoselective version [96,97,98,99] of the above-described reaction, using a chiral auxiliary (Scheme 90).

Oppolzer’s camphorsultam was chosen as an auxiliary, while the reactions were tested in SP (using Wang resin) and in solution. The oxime was attached to a carboxylic acid derivative which, in the SP version, served as the spacer between the reactive moiety and the polymeric support (compound **267**). In a first attempt, it was decided to try ethylation of **267** following the method described in Scheme 88, using Et_3_B or Et_2_Zn at low temperatures (-78 °C) in order to facilitate stereoselectivity. In both cases the results were very promising, high yields (59-74%), with little variation between solvents (DCM slightly better than toluene) and radical source (Et_3_B slightly better than Et_2_Zn), with diastereoselectivity higher than 95%. In order to prove the general validity of the method, radical addition from a different radical source was tried. At first Bu_3_SnH was used together with an alkyl iodide, but results were disappointing: low yield (around 40%) and a mixture of products were obtained (addition of ethyl radical prevailed). When the reaction was run without the tin hydride, yield improved (78%), but the great majority was still the undesired product (5:1), which was in contrast with the analogous solution-phase reaction. In order to overcome these problems, the conditions were changed: the reaction was performed using a mixture of R-I (30 eq.) and toluene (4:1) under iodine atom transfer conditions in the presence of Et_3_B or Et_2_Zn (5 eq.), and at higher temperatures (0 °C). This led to higher yields (R = *i*-Pr: 69% Et_3_B, 53% Et_2_Zn; R = *c*-hexyl: 58% Et_3_B, 41% Et_2_Zn), and higher stereoselectivities (>90%) compared to the in-solution reactions. The reason for the presence of such large amount of by-product in SP was not explained, however, this tendency increased at lower temperatures, suggesting that triethylborane concentrate on solid-support as Lewis acid, releasing a large amount of ethyl radical around the surface of the polymer.

**Scheme 91:**
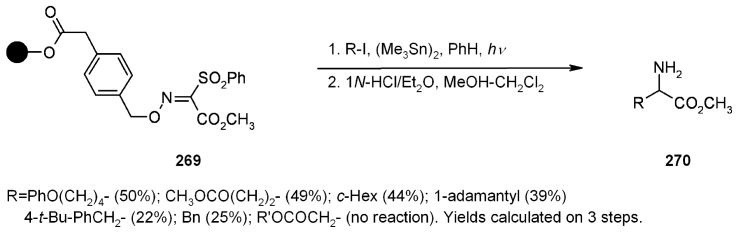
Kim’s radical addition to supported oxime ether.

In an approach similar to the one described by Naito in Scheme 89, Kim and colleagues [100] performed the synthesis of α-aminoesters by addition of alkyl radical to an anchored phenylsulfonyl oxime ether (Scheme 91).

The reaction was successful for unactivated alkyl radicals, lower-yielding in the case of addition of benzyl radicals (either from benzyl iodide or bromide), while no reaction was observed for α-carbonyl radicals, which suggests that the process is governed by radical philicity.

### 3.2. Carbon-Carbon Bond Formation through Intramolecular Reactions

#### 3.2.1. Radical Addition to Oximes

**Scheme 92:**
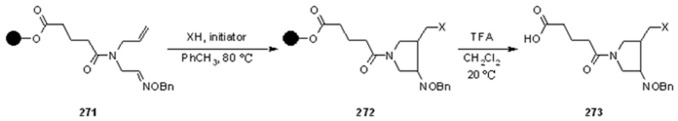
Naito’s SP-atom transfer radical cyclization into oxime-alkene.

**Scheme 93:**
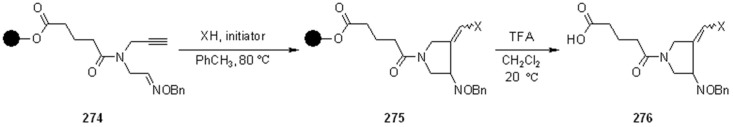
Naito’s SP-atom transfer radical cyclization into oxime-alkyne.

In successive papers [96,97,101,102,103], Naito’s group continued to study the reactivity of oxime ethers on solid-support by testing a 5-*exo*-trig radical cyclization for the synthesis of pyrrolidines (Scheme 92 and Scheme 93).

**Scheme 94:**
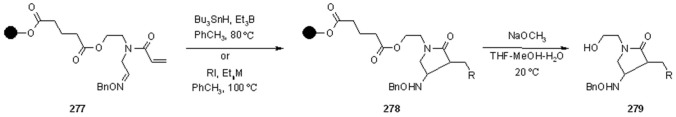
Radical addition to an electron-poor alkene.

**Scheme 95:**
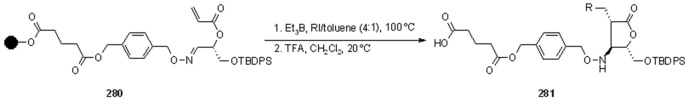
Example of stereoselective addition/cyclization reaction.

Resins **271** (Scheme 92) and **274** (Scheme 93) were prepared and reacted to test their cyclization potentiality. The major difference between the reactions depicted in Scheme 89, Scheme 90, Scheme 91 was failure of cyclization at low temperature. For this reason, it was necessary to use toluene as solvent, heated up to 80 °C. The use of Bu_3_SnH as XH gave reasonable yield when AIBN was used as radical initiator (Scheme 92: 47%). Better results were obtained using Et_3_B (Scheme 92: 64%; Scheme 93: 77%), while further variation of the radical initiator did not improve the process: in fact, 9-BBN gave only trace of product and Et_2_Zn gave successful cyclization, but low yield (Scheme 92: 26%). Use of TTMSS in place of Bu_3_SnH gave lower yield (Scheme 92: 50%). As observed previously (see Scheme 89), the desired reaction is always in competition with ethyl radical addition-cyclization and when a less reactive radical precursor such as Et_3_SiH was used, the sole product was X = Et. The same reaction was successfully tried on an appropriate substrate containing a solid-supported α,β-unsaturated amide (Scheme 94) and extended to check the possibility of stereoselection using an asymmetric substrate (Scheme 95).

The radical addition to an electron-poor alkene is shown in Scheme 94. In a first attempt the addition of a stannyl radical was tested and product **279** was recovered after cleavage in good yield (64%). The same reaction was then run under iodine-atom transfer conditions, using different R-I. The results were satisfying, although a large amount of alkyl iodide had to be used (30 eq.). The only reaction that failed was the one with the bulky *t*-Bu radical (R = *i*-Pr: 69%; *c*-hexyl: 54%; *c*-pentyl: 59%; *s*-Bu: 55%). The results obtained using Et_n_M without R-I were rather interesting, thus, when Et_3_B was used, a 72% of **279** product (R = Et) was isolated, while the use of Et_2_Zn gave only a 12% of the same product.

Scheme 95 displays the stereoselective addition/cyclization reaction promoted by alkyl radicals, using asymmetric substrate **280**. The reaction was run in a strong excess of R-I (4:1 v/v with toluene), because, with smaller amounts, the product of ethyl radical addition was predominant. The products **281** were recovered in reasonable yields (R = *i*-Pr: 57%; *c*-hexyl: 50%; Et: 92%, when the reaction was run with only Et_3_B), but with a constant diasterometic ratio of 8:1, independently of the nature of the adding radical.

#### 3.2.2. Radical Addition to Alkenes, Alkynes and Arenes

**Scheme 96:**
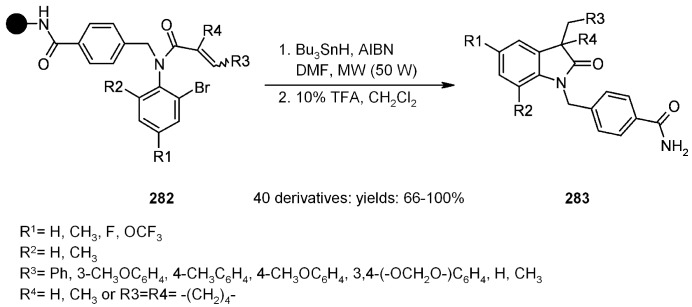
Fukase’s SP-radical cyclization into alkenes.

A radical cyclization for the synthesis of 2-oxindoles was studied by Fukase’s group [104]. In this case, *N*-(2-bromophenyl)acrylamides were anchored to the SP and treated under classical radical conditions (Bu_3_SnH, AIBN) (Scheme 96).

Oxindoles such as **283** were obtained smoothly under MW irradiation (<1 h), compared to conventional heating (24 h). Interestingly, the reaction was run in DMF, a solvent where Bu_3_SnH is rather insoluble. This characteristic forced an increase in concentration of the *H*-donor on the surface of the resin. In addition, the same reaction run in solution gave no product at all, sustaining the thesis of concentration effect [105].

**Scheme 97:**
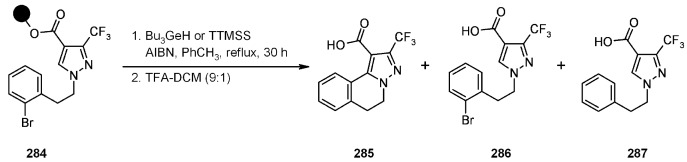
Bowman’s SP-radical addition into pyrazole.

Bowman and coworkers [106] studied the addition of reactive aryl radicals to azoles and extended the methodology to SP using less toxic and troublesome TBGH and TTMSS (Scheme 97).

The probe scaffold was anchored to a Wang resin. The results were rather disappointing compared to the relative reactions carried out in solution: in fact, when TBGH was used only 20% of **285** was isolated after cleavage, together with 70% of unreacted **286**. However, switching to TTMSS improved the yield of **285** (53%), but this time, the side product was the reduced, uncyclized **287** (27%).

**Scheme 98:**
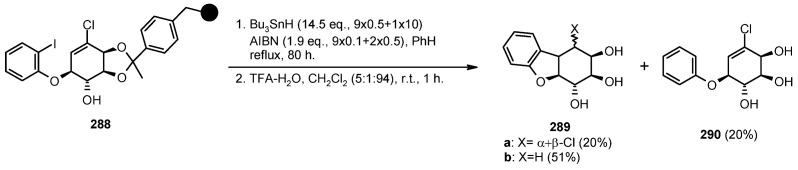
De Maesmaeker’s SP-aryl radical cyclization into alkene.

**Scheme 99:**
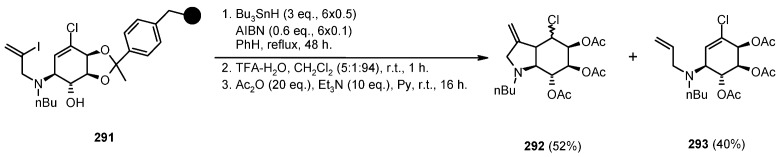
De Maesmaeker’s SP-vinyl radical cyclization into alkene.

De Maesmaeker and Wendeborn [107] studied the functionalization of cyclohexendiol derivatives *via* radical cyclization on solid phase. Cyclization was performed using an aryl radical (Scheme 98) and a vinyl radical (Scheme 99), giving in both cases a mixture of products.

In the first case (Scheme 97), product **289a** was obtained as the minor component together with the prematurely reduced starting material **290**, and the product of over-reduction of **289a** (**289b**, X = H). Unfortunately, it resulted impossible to achieve complete removal of the chlorine atom without increasing the amount of **290**. In the second reaction (Scheme 98), a large amount of reduced product **293** was found, while chlorine atom resisted in the desired product **292**.

**Scheme 100:**
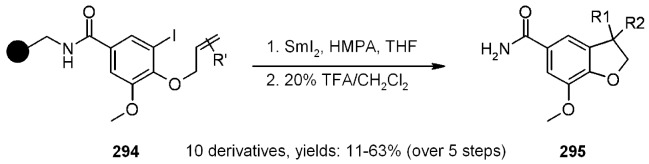
Armstrong’s SP-aryl radical cyclization into alkenes.

Du and Armstrong [108] provided a milder approach to the synthesis of solid-supported benzofurans via radical cyclization, which is based on the use of SmI_2_ (Scheme 100).

**Scheme 101:**
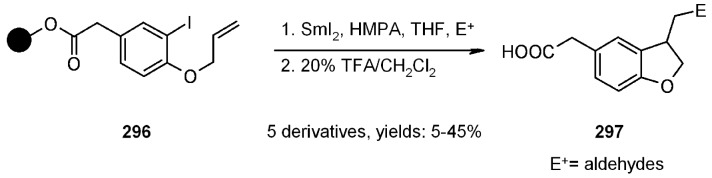
Example of radical cyclization/electrophile capture.

Yields were measured over 5 steps (4 of them necessary to synthesize **294**). The reaction was run at room temperature and a range of diverse substituted alkenes was tested. The cyclization reaction took place in almost all cases, being the α,β-unsaturated ester (**294** with R’ = COOEt) derivative an exception. However, in some cases, the yields were lower due to a variable amount of disproportionation product. This side-reaction proved to be substrate-dependent more than SP-dependent, since parallel test reactions in solution were carried out, revealing similar problematic. A major drawback of this approach could be the use of toxic reagents in excess (HMPA was necessary for the reaction to take place) and the difficulty in washing out the samarium ions when ungrafted polystyrene resin (Rink) was used. The latter problem was resolved using polyethylene glycol grafted resin, which swells well in aqueous solvents, making wash-up with NaHCO_3_ saturated solution more efficient for the elimination of Sm^3+^ impurities from the beads. In a subsequent paper [109], the same group brought forward the reaction depicted in the previous scheme by capture of the anionic metal derivative, formed after cyclization, with an electrophile (Scheme 101).

The electrophiles chosen were aldehydes, using reaction conditions similar to those described earlier. As reference, the test reactions were also run in solution, where it was acknowledged the short half-life of the anion formed. 

Compared to the previous example, attachment to the SP was performed *via* an appropriate handle on the benzene ring. In a first attempt Rink resin was used, giving no product of electrophilic quenching of the anion. Since the amide nitrogen in the linking group of Rink was supposed to be responsible for early interception of the anion generated after radical cyclization, it was decided to use the ester linkage of TentaGel S BHP resin (a resin also swelling better in the solvent used for the reaction). In this case, interception was effective, even if in a mixture with simple cyclized product. Since the yields were strongly dependent on the electrophile nature, this method did not present the quality necessary to be used in combinatorial chemistry.

**Scheme 102:**
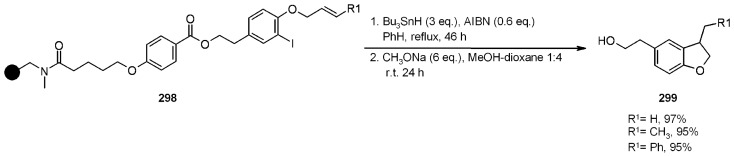
De Maesmaeker’s SP-aryl radical cyclization into alkenes.

Following the same idea, Berteina and De Maesmaeker [110] tested the 5-*exo*-trig cyclization to form 2,3-dihydro-benzofurans. Either the aryl iodide or the unsaturated counterpart, tethered by means of an oxygen atom, was directly anchored to aminomethylphenyl functionalized polystyrene beads. Usually, a spacer was inserted between the resin and the reactive molecule. As first attempt, the radical cyclization was performed under usual radical conditions (Scheme 102).

The radical cyclization depicted in Scheme 102 was effective, high yielding and no product of early reduction or starting material was found. 

**Scheme 103:**
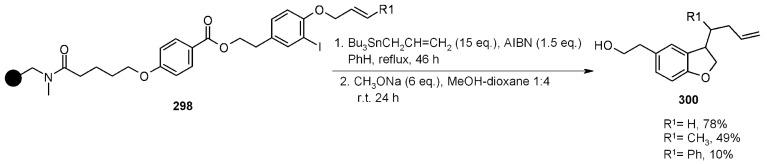
Example of radical cyclization/allyl interception.

Interception of the alkyl radical formed after cyclization with allytributyltin was also attempted (Scheme 103). For R^1^ = H, the reaction gave a good yield of desired product **300** (beside 22% of simple cyclized product **299**), while for R^1^ = CH_3_ or Ph, yields were pretty low and after cleavage the main product was found to be the unreacted starting material (beside 10% of **299**), demonstrating a slower reaction that can be explained on the basis of steric hindrance.

**Scheme 104:**
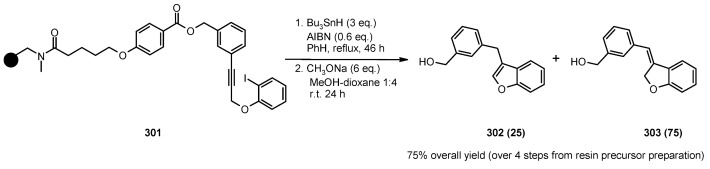
De Maesmaeker’s SP-aryl radical cyclizatino into alkyne.

In a second attempt, it was chosen to have the unsaturated moiety, in this case an alkyne, directly connected to the SP. The reaction led to a mixture of two products **302** and **303** (Scheme 104).

**Scheme 105:**
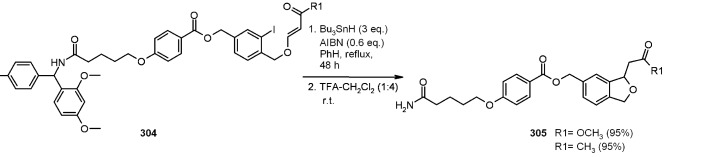
De Maesmaeker’s synthesis of benzofurans **305**.

In another publication [111], the same group used a similar 5-*exo*-trig cyclization to form 1,3-dihydro-isobenzofurans (Scheme 105).

The acid-labile Rink linker was necessary since, following the strategy adopted in Scheme 103 and Scheme 104, low yields of desired products (due to partial decomposition under the basic cleavage conditions) were obtained. This change of plan gave almost quantitative yields after cleavage.

**Scheme 106:**
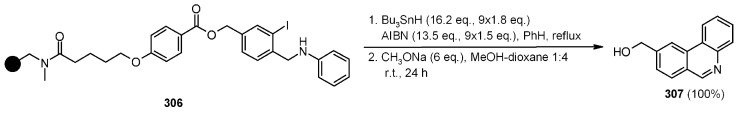
De Maesmaeker’s SP-aryl radical cyclization into a benzene ring.

In another experiment, it was evaluated the radical cyclization onto an aromatic ring, which yielded a benzoquinoline (Scheme 106).

In this case, it was necessary to use large amounts of *H*-donor and, most importantly, of radical initiator (AIBN), added in aliquots during the reaction. Lower amounts of the reagents gave uncompleted conversions. The need of a large excess of AIBN is probably due to its role played in the aromatization step after cyclization. 

**Scheme 107:**
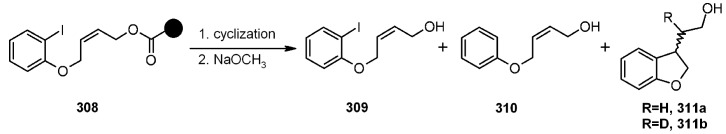
Routledge’s SP-aryl radical cyclization into alkene.

Also Routledge’s group [112] used the intramolecular radical cyclization to form tetrahydrofurans derivatives (such as the one depicted in Scheme 100) testing the use of hypophosphite salts as radical carriers in alternative to the toxic TBTH on SP (Scheme 107).

**Scheme 108:**
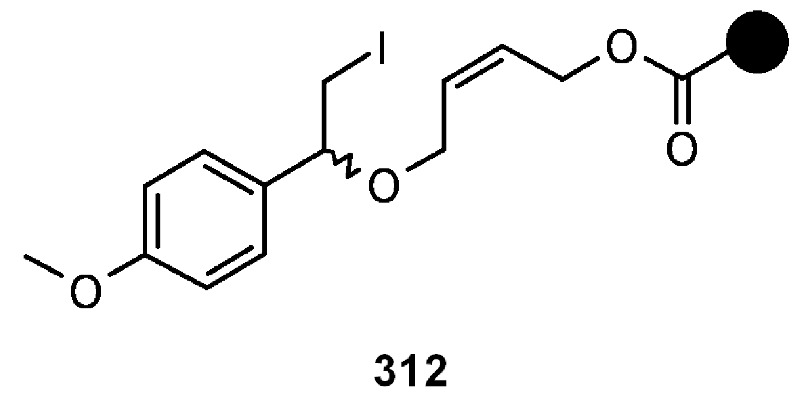
Structure of alkyl radical precursor **312**.

As reference, the reaction was first run using TBTH (10 eq., toluene, reflux, 4 h) and AIBN (1 eq.), on a 2% divinylbenzene cross-linked carboxypolystyrene resin, giving a 95% yield of cyclized product **311a** (beside 4% of **309** and 1% of **310**). The reaction was then run using *N-*ethylpiperidine hypophosphite (EPHP, 20 eq.) and AIBN (3 eq.), but at best, only a 57% yield of **311a** was isolated (besides 38% of **309** and 5% of **310**). The reason for this behavior was initially supposed to lay in the nature of the solvent used (toluene), since EPHP is a salt and its concentration on the surface of the resin could be low in apolar solvents. However, when the reaction was run in polar mixtures (THF-EtOH 4:1), no real change of product distribution was observed (63% **311a**, 31% **309**, 6% **310**). The attention was then shifted to the nature of the solid phase and a range of cross-linked and PEG grafted solid-supports was tested. The idea was that resins more hydrophilic than polystyrene would allow the radical carrier to approach the solid surface. For the purpose the following resins were used: NovaSyn^®^ TG carboxy resin, a macroporous resin (ArgoPore^®^), PEG grafted resins, HypoGel^®^ and ArgoGel^®^ and the polytetrahydrofuran cross-linked resin, *J* anda*J*el^®^. The best results were obtained using the latter, which gave a 98% of **311a** (beside a 2% of **310**), under optimized conditions (20 eq. EPHP, initiator 2 eq., THF-EtOH 4:1, 48 h, under reflux). To confirm the validity of the choice made, a different reaction was carried out, using a primary alkyl radical precursor **312** (Scheme 108). 

The reaction was successful, with a 92% yield of desired cyclized product. The use of alternatives such as EPHP on SP could allow radical chemistry to become a more useful method for SP synthesis, with potential extension to combinatorial and medicinal chemistry.

In a recent paper [113], the same group studied the influence of microenvironment effects in the reaction depicted in Scheme 107 by determination of deuterium incorporation in the cyclized product. In order to do so, cyclization was carried out using deuterated EPHP or Bu_3_SnD in deuterated solvents (d_8_-THF: d_6_-EtOH 4:1 or 1:1; d_4_-MeOH; d_6_-benzene). The ratio of deuterated product **311b** (R=D) versus **311a** (R=H) was measured by GC-MS analysis.

The objective was the rationalization of the role played by the polymer backbone/linker in the radical *H*-abstraction, a side-reaction encountered in radical chemistry on SP. A first conclusion was that, as already observed by Curran [8], all resins can compete with relatively slow *H*-transfer reactions (such as *H*-abstraction from EPHP by an alkyl radical). However, the overall picture indicated that there are multiple factors influencing radical chemistry on SP: nature of the resin and linker (flexibility), solvent (swelling properties) and nature of the radical carrier (bond strength and solubility). Therefore, a general prediction of the effects of a specific resin on kinetics and mechanism of a radical reaction is rather difficult and the combination of the above-mentioned factors need to be considered case-by-case.

**Scheme 109:**
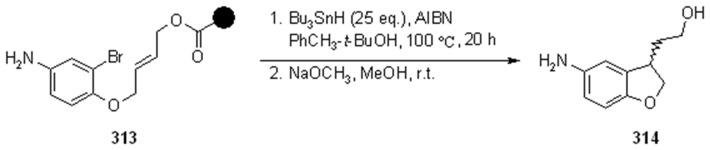
Balasubramanian’s SP-aryl radical cyclization into alkene.

**Scheme 110:**
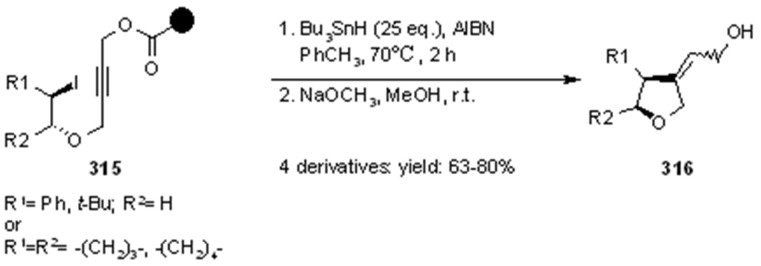
Balasubramanian’s SP-alkyl radical cyclization into alkene.

A more traditional approach was used by Balasubramanian and coworkers [114], who performed aryl radicals cyclization into alkenes to prepare benzofuran derivatives (Scheme 109) and alkyl radicals cyclization into alkynes to prepare functionalized furans (Scheme 110). The reactions were run using a very large excess of the *H*-donor, Bu_3_SnH. When the same excess of organostannyl hydride was used in solution, large amount of early reduction products were normally obtained. Therefore, somehow, use of the solid phase seemed to neutralize this inconvenience.

Both carboxylated PS and TentaGel-COOH were used as resins. The main difference among them was the amount of radical initiator needed; in fact, in the former case, an excess of AIBN had to be used, while in the latter, only 6% mol was sufficient to perform complete conversion. The reason for this different behavior was attributed to chain-terminating processes involving the benzylic hydrogen on the polystyrene backbone, absent in the TentaGel resin. The lack of reduced side-products was tested on a slower (three orders of magnitude) reaction (shown in Scheme 110) usually more sensitive to early reduction, but even in this case it was not observed.

**Scheme 111:**
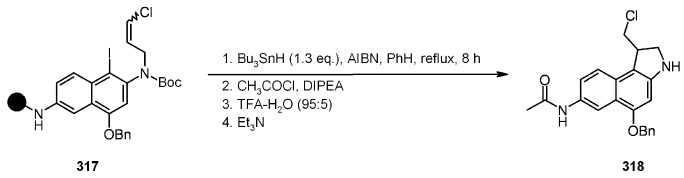
Lown’s use of SP-aryl radical cyclization for the synthesis of seco-CBI.

In an application to the synthesis of molecule of biological interest, a 5-*exo*-trig radical cyclization was performed by Lown’s group [115] for the synthesis of 1-chloromethyl-1,2-dihydro-3H-benz[*e*]indole (seco-CBI) and a polyamide conjugate (Scheme 111).

**Scheme 112:**
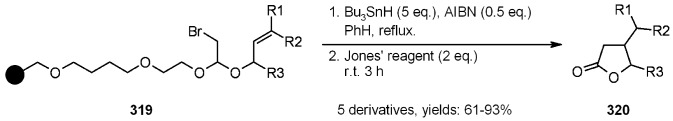
Toru’s SP-alkyl radical cyclization into alkenes.

The radical reaction was carried out starting from bromo-Wang resin according to the procedures applied in solution, commonly used for the synthesis of CC-1065 derivatives. To measure the efficacy of the reaction on SP, the product of cyclization was first acetylated and then cleaved, to yield a total 74% (48% plus a 26% of the product with the Boc protecting group still in place). The synthesis of a bis-polyamide-*seco*-CBI conjugate was completed using **318** and performing additional amide couplings both on solid- and solution-phase. Toru and colleagues [116] performed the intramolecular radical addition of an alkyl radical to unsaturated *C-C* bonds, in order to form γ-butyrolactones (Scheme 112).

Merrifield resin was functionalized with an appropriate linear spacer and radical precursors **319** were prepared on the SP by addition of allyl (or propargyl) alcohols to the loaded vinyl ether, in the presence of NBS. The radical reaction was performed under common conditions and the excess of H-donor did not give uncyclized, reduced side-products. The toxic organotin by-products were separated by filtration and washing, while cleavage of the desired products from the resin was achieved using Jones’ oxidation. Yields were rather satisfactory, even when a 5-*exo*-dig cyclization on a triple bond was tested (47%, *E*:*Z* = 1:9). 

**Scheme 113:**
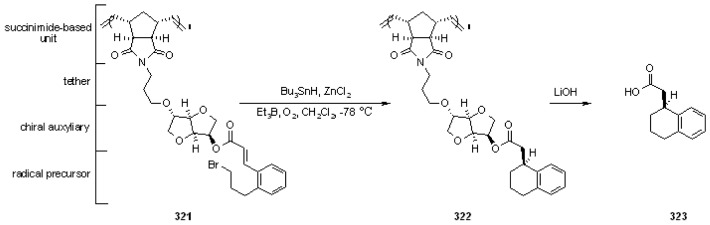
Enholm’s stereoselective alkyl radical cyclization on ROMP polymer.

During his studies towards the use of supported radical chemistry, Enholm [117] proposed the first example of stereoselective radical cyclization on a soluble support. The use of soluble supports (precipitating at –78 °C in MeOH) represent a great advantage for SP radical reactions since, according to the authors, the rates of the reactions involved are similar to those run in solution. Thus, a custom polymer incorporating a chiral auxiliary unit was synthesized by ring opening metathesis polymerization (ROMP). The radical reaction studied was a 6-*exo*-trig cyclization (Scheme 113).

After building the monomer, the polymer was prepared employing a Grubbs-type ruthenium-based catalyst. Each monomer contained a succinimide anchor tethered to an (+)-isosorbide unit (the chiral auxiliary) esterificated with the radical precursor. After the ROMP, the polymer obtained reacted under radical conditions in the presence of a Lewis acid. Subsequent cleavage with lithium hydroxide afforded the desired acid **323**. Constructing the polymer in this way, it was possible to achieve a 100% loading, a result not viable using commercial resins. The results were encouraging since, beside an 80% isolated yield, there was a >99% ee (being ZnCl_2_ the best solution). The use of a combination of low temperatures and Lewis acid for stereoselective radical cyclization is known [118,119,120,121,122,123], however, in this case, the use of zinc chloride proved to be far more better than other LA (such as MgBr_2_ or Yb(OTf)_3_), probably owing to the presence of the polymer-embedded sugar which creates a favorable environment for zinc-mediated stereoselection.

**Scheme 114:**
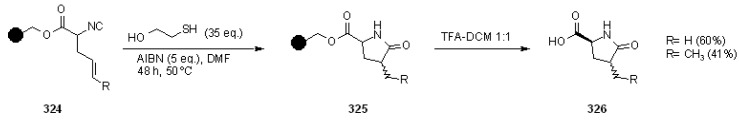
Kilburn’s thiol-mediated radical cyclization into SP-isocyanides.

Kilburn’s group [124] exploited the reactivity of isocyanides to perform thiol-mediated radical cyclizations to obtain pyrroline or pyrrolidinone derivatives. In Scheme 114 a Wang-type resin was used. Treatment under radical cyclization conditions in the presence of 2-mercaptoethanol gave resin **325** that after trifluoroacetic acid-mediated cleavage gave pyrrolidinone **326** in reasonable yields.

**Scheme 115:**
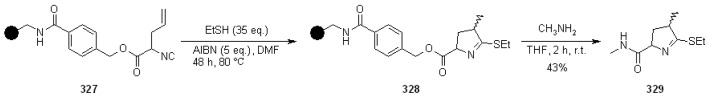
Kilburn’s pyrroline synthesis.

When HMBA-AM resin was used (Scheme 115), the use of ethanethiol during the cyclization step gave rise to resin **328**, which could be cleaved under nucleophilic conditions preserving the pyrroline structure to yield amide derivative **329**. In both cases the starting isonitrile was built on resin, which has the advantage of avoiding the handling of the unpleasant substance.

**Scheme 116:**
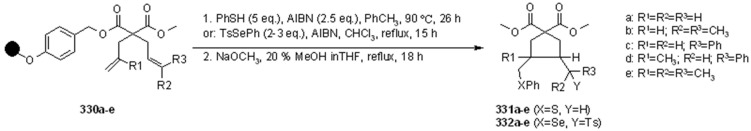
Harrowven’s SP-atom transfer radical cyclization into alkenes-alkenes.

Harrowven and colleagues [125] reported two new methods for radical cyclization on SP using supported dienes and, respectively, thiyl and tosyl radical. In this case heating of the loaded resin in the presence of AIBN and the radical precursor (PhSH or TsSePh) in an appropriate solvent was sufficient (Scheme 116).

Radical precursors **330a-e** were loaded on a PS-Wang resin. The results obtained when using phenylthiol (X = S, Y = H) as radical source/cyclization promoter were rather satisfactory, with yields of **331** ranging between 69-78%; when using *p*-tolyl benzeneselenosulfonate (X = Se, Y = Ts) on the same substrates, the desired products **332** were recovered in comparable yields (66-74%). The isomeric product distribution followed Beckwith’s guidelines for radical cyclizations [126,127].

### 3.3. Carbon-Heteroatom Formation

**Scheme 117:**
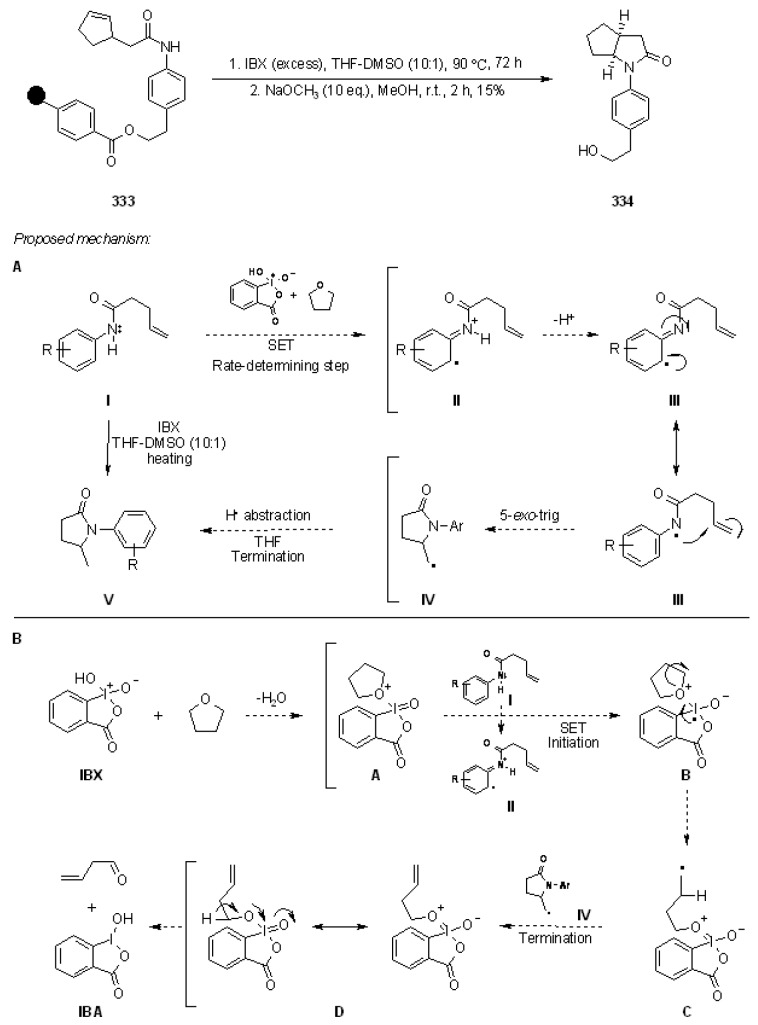
Nicolaou’s IBX-promoted SP-radical cyclization.

An interesting radical cyclization initiated by IBX was discovered [128,129,130] and thoroughly studied by Nicolaou. Mechanism and limits of the reaction as well as an application in solid-phase were subsequently reported [131] (Scheme 117). The interesting mechanism comprises a radical reaction initiated by IBX (A), where THF actively participate (B) and explains the reason for the need of an aryl amide for the success of the reaction. While it was tested and performed with good results in solution, the extension to the SP resulted in poor yield of the desired product (15%), probably due to oxidation of the resin by the IBX excess.

**Scheme 118:**
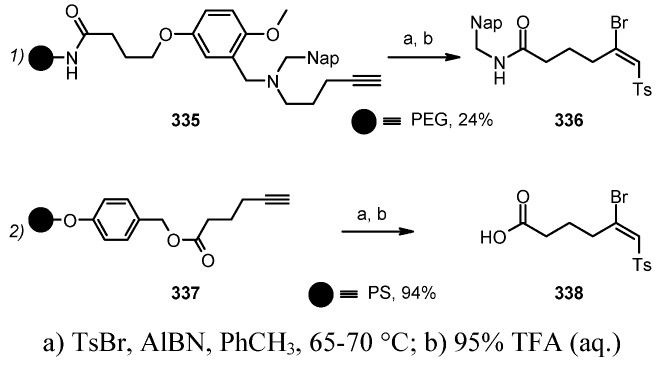
Caddick’s radical addition to alkynes.

**Scheme 119:**
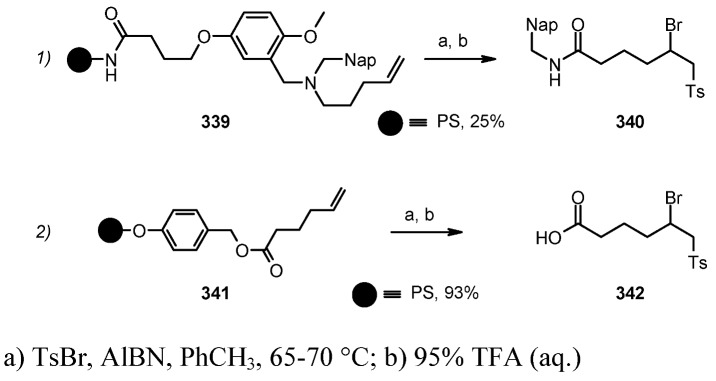
Caddick’s radical addition to alkenes.

Caddick and coworkers performed the addition of sulfonyl radicals to isolated alkenes (Scheme 118) and alkynes (Scheme 119) [132] and the best conditions are displayed below. Interestingly, the nature of solvent, linker (eq. *1*,*2* of Scheme 118 *vs.* eq. *1,2* of Scheme 119) and resin did have great influence on the reaction outcome, spanning between combinations were there was no reaction at all, to conditions giving decomposition.

**Scheme 120:**
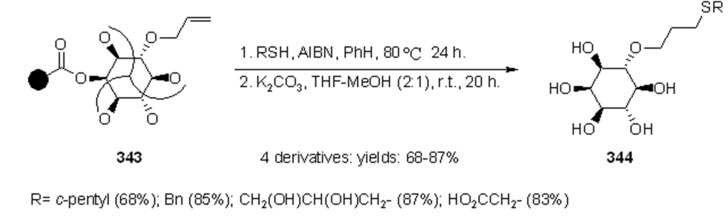
Plourde Jr.’s radical addition to supported alkenes.

**Scheme 121:**
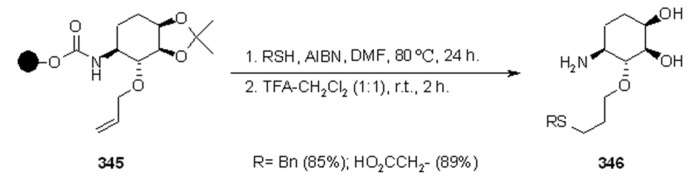
Example of Plourde Jr.’s use of carbamate linker.

During the studies about the transformation of hydrophilic to hydrophobic groups, in order to change the pharmacological properties of molecules, Plourde Jr. [133] explored the intermolecular radical addition of thiols to supported alkenes (Scheme 120 and Scheme 121).

The reactions were run under the usual radical conditions, even if when the carbamate linker was used (Scheme 121) an uncommon solvent for radical reactions such as DMF was chosen. Yields were generally good, however, when R = Ar or HetAr, no reactions was observed (not shown). Addition of a thiol-bearing resin to an alkene was employed also for the synthesis of a new linker useful for the synthesis of amines. In order to improve the scope of the 2-(thiobenzyl)ethyl carbamate linker normally used for such a purpose, Timár and Gallagher [134] proposed to start from Merrifield SH resin.

**Scheme 122:**
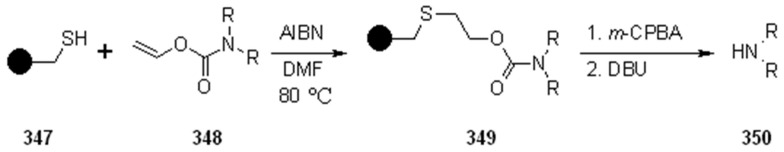
Gallagher’s SP-radical synthesis of amines.

This (Scheme 122) was treated with the previously prepared *N*-vinyloxycarbonyl derivative **348**, under radical conditions. Addition of the benzyl thiyl radical to the vinyloxy moiety loaded the desired starting material to the resin, giving **349**, which was treated under oxidative/basic conditions for cleavage. This modification offers an entry for secondary amines, complementing the original procedure (exploiting the reaction between a supported alcohol and an isocyanate), only viable for primary amines.

**Scheme 123:**
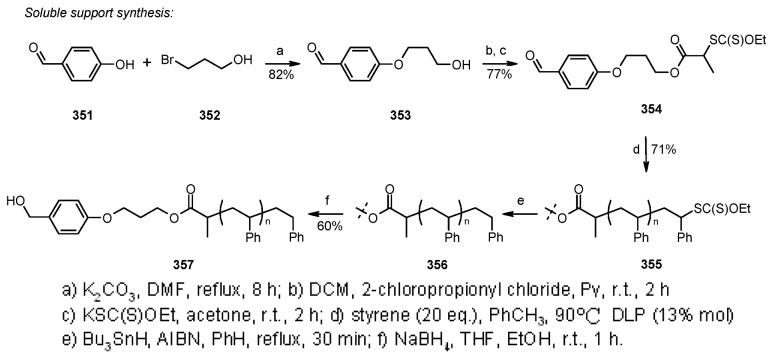
Zard’s synthesis of soluble support for radical cyclization.

A possible problem in SP intermolecular radical reactions consists of the lower rate constants compared to the reactions run in solution, probably due to the heterogeneous nature of SP reactions. Zard’s group [135] tried to address this problem by synthesizing a Wang-type soluble support. This support was then compared with the classical polystyrene Wang resin, *via* the radical addition of supported xanthates to olefins in solution and by the addition of immobilized olefins to free xanthates. Soluble support **357** was synthesized by xanthate polymerization and employed for intermolecular test radical addition. (Scheme 123)

**Scheme 124:**
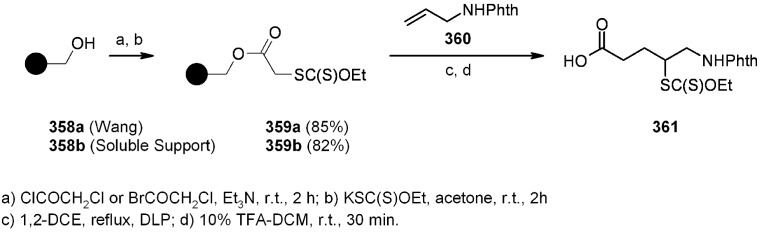
Zard’s use of supported xanthates.

In Scheme 124 the supported xanthate was reacted with an excess olefin **360** under tin-free radical conditions. For both resins, the reaction was biased by the formation of by-products, compared to the one run without exploiting polymers. However, **359b** (soluble support) gave cleaner reaction profile and higher yields (54% *vs.* 26%) compared to **359a** (Wang). For Wang resin a large excess of olefin **360** and 30% mol of lauroyl peroxide (DLP) needed to be used, while for the soluble support, a 3-fold excess of **360** and 40% DLP were needed to complete the reaction. 

**Scheme 125:**
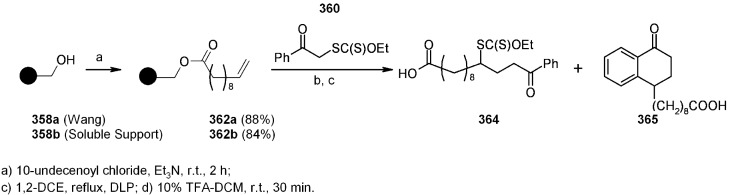
Zard’s use of supported alkenes.

When the reagents were inverted (Scheme 125), in both cases, the reaction did not go to completion and side-product **365** was found in similar proportion to the desired product. Despite this, the reaction was substantially cleaner compared to the one described in Scheme 124, even if it was still needed a consistent amount of DLP (50% mol) compared to the polymer-free reaction. Final yields were reasonable (52% for Wang, 70% for soluble support). The presence of side product **365** says that at certain xanthate concentrations, the intermolecular reaction is in competition with the intramolecular process, preventing the reaction to go forward in the desired direction. These results suggest that intermolecular radical processes are viable on SP and that the use of soluble support has more benefits compared to the heterogeneous classical SP synthesis.

**Scheme 126:**
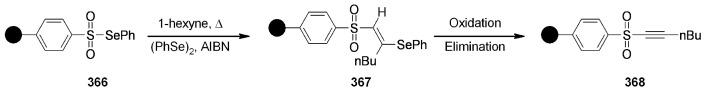
Back’s SP-radical addition of resin **366** to free alkynes.

Back and Zhai [136] performed a radical addition of PS-arylselenosulfonates to alkynes (Scheme 126).

**Scheme 127:**
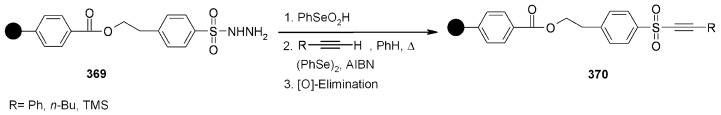
Back’s SP-radical addition of resin **369** to free alkynes.

Acetylenic sulfones of the kind of **368** (or **370**) are useful synthones for a variety of reactions (such as cycloadditions). Their first attempt to perform their synthesis on SP through radical addition to 1-hexyne is displayed in Scheme 127. Radical reaction and subsequent oxidation-elimination to restore the triple bond were effective, however, when **368** was further reacted under cyclization conditions, it did not yield any product. For this reason, it was added a spacer to the SP, therefore, resin **369** was prepared and reacted to yield products **370**. Finally, **370** were successfully reacted under cyclization conditions, giving the desired products in good yields.

**Scheme 128:**
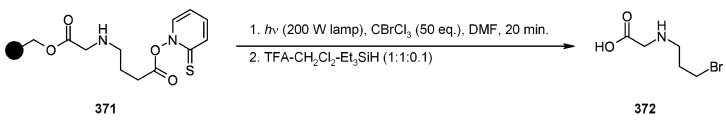
Taddei’s SP-synthesis of unnatural AAs by radical approach.

In the quest for new methodologies viable for the synthesis of small molecules on SP, Attardi and Taddei [137] presented a radical approach for the construction of unnatural amino acids and their small oligopeptides (Scheme 128).

In the example reported above the Wang resin anchored Barton ester **371** was obtained by coupling of the correspondent acid with 1-hydroxy-2-pyridinethione. The photolabile product was submitted to irradiation to give radical fragmentation and loss of CO_2_, followed by trapping with CBrCl_3_. To minimize side-products it was necessary the use of DMF as solvent and 50 equivalents of the halogenated chemical reagent. After cleavage, a respectable 72% yield was obtained. The same process was applied to di- and tri-peptides, while the resulting alkylbromides were further reacted with nucleophiles to obtain the desired unnatural aminoacid side-chain.

### 3.4. Other

**Scheme 129:**
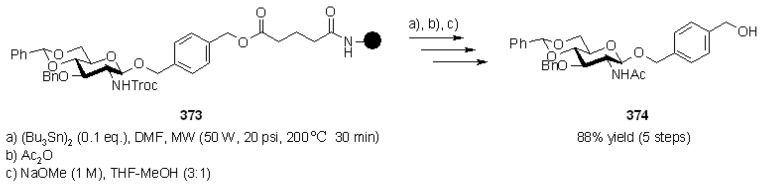
Fukase’s SP Troc radical deprotection.

Troc (2,2,2-trichloroethoxycarbonyl) group is a widely used protecting group in organic, especially oligosaccharide, chemistry. Since its removal is often performed under heterogeneous conditions, it is not ideal for SP chemistry. Fukase [105] studied a methodology for Troc removal on a substrate anchored to SP, performed using a radical methodology (Scheme 129). The conditions used were tested in solution before extension of the methodology to SP and it was found that DMF was the best solvent (the reaction did not proceed in toluene), probably due to a concentration effect of the organotin derivative to the polymer support; and that the reactions proceeded slowly under conventional heating, therefore, microwave irradiation seemed to be the best choice. Since the amount of (Bu_3_Sn)_2_ was substoichiometric, an involvement of DMF in the catalytic cycle was also suggested. Beside the high yields obtained, the method was also effective because of the absence of dichloroethoxycarbonylated byproducts. 

**Scheme 130:**
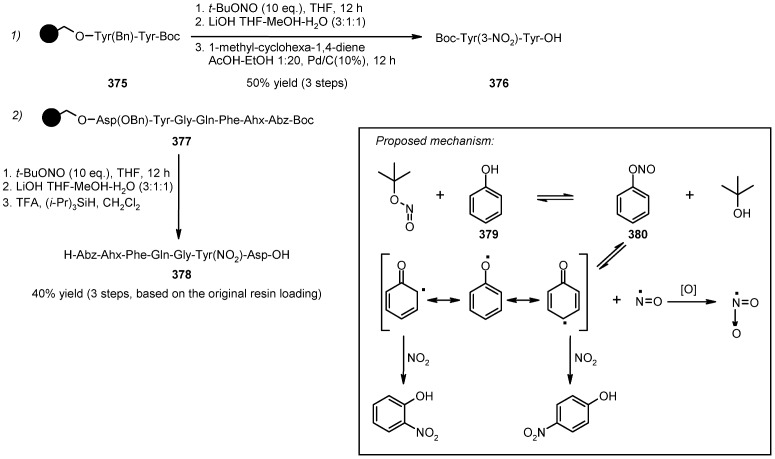
Savinov’s nitration of phenols.

The couple 2-aminobenzoic acid (Abz) and 3-nitrotyrosine (Tyr(3-NO_2_)) is used as fluorophore-quencher combination in FRET (Fluorescence Resonance Energy Transfer) methodology for monitoring structural, functional, or aggregation changes in evaluated biomolecules. Since the couple can be easily incorporated in peptides, it would be of great advantage having a method for selective nitration of tyrosine incorporated on a peptide anchored to SP. Savinov and colleagues [138] did find this very mild, SP-friendly methodology for selective nitration of phenols. After a careful optimization of the reaction conditions and a mechanistic study, the reaction was tested on resin-bound substrates (Scheme 130).

The right solvent for the reaction (THF) was first identified in solution, where some evidence of chemoselectivity was also found. Most importantly, the reaction worked only when a phenol hydroxyl group was present. Subsequently, the protocol was tested on dipeptide **375**, confirming the need for a free phenolic hydroxyl group and, finally, on a peptide such as **376**, giving the desired nitrotyrosine derivative **378** in good yield. The hypothesized mechanism was confirmed by the isolation of the *O*-nitroso derivative (such as **380**) and the observation of the stabilized phenoxyl radical of **379**, with its characteristic blue color. Decomposition of an *O*-nitroso derivative such as **380** led to nitric oxide, which underwent oxidation (probably from air oxygen) to nitrogen dioxide that subsequently coupled with the phenoxyl radical to give the nitration products. It is clear from the proposed mechanism, that the phenol hydroxyl group participates in the reaction, in fact, when anisole was subjected to the same experimental conditions, no nitrated products were observed. 

## 4. Conclusions

Radical chemistry, despite having a long-standing tradition and deep relationships with polymers, has become one of the available means for solid-phase synthesis only in relatively recent years. Nevertheless, the wealth of the examples we have provided above witnesses the fruition of such a merging. 

On the other hand, in the same period of time the concept of diversity-oriented synthesis (DOS) [12] has abruptly emerged in organic chemistry, with the goal of providing collections of small molecules. A number of tools are available to achieve such an end and in this context, as this review has illustrated, radical chemistry applied to SP represents an important and valid alternative.
